# Advancing understanding of *Ficus carica*: a comprehensive genomic analysis reveals evolutionary patterns and metabolic pathway insights

**DOI:** 10.3389/fpls.2023.1298417

**Published:** 2023-12-07

**Authors:** Yuting Bao, Miaohua He, Chenji Zhang, Sirong Jiang, Long Zhao, Zhengwen Ye, Qian Sun, Zhiqiang Xia, Meiling Zou

**Affiliations:** ^1^ Sanya Nanfan Research Institute of Hainan University, Hainan Yazhou Bay Seed Laboratory, Sanya, China; ^2^ College of Agriculture, China Agricultural University, Beijing, China; ^3^ Academy of Agriculture and Forestry Sciences, Qinghai University, Xining, Qinghai, China; ^4^ Forestry and Fruit Research Institute, Shanghai Academy of Agricultural Sciences, Shanghai, China; ^5^ College of Life Science and Technology, Guangxi University, Guangxi, China

**Keywords:** *Ficus carica*, chromosome evolution, genome, FhAG2, CHS

## Abstract

*Ficus carica* L. (dioecious), the most significant commercial species in the genus *Ficus*, which has been cultivated for more than 11,000 years and was one of the first species to be domesticated. Herein, we reported the most comprehensive *F. carica* genome currently. The contig N50 of the Orphan *fig* was 9.78 Mb, and genome size was 366.34 Mb with 13 chromosomes. Based on the high-quality genome, we discovered that *F. carica* diverged from *Ficus microcarpa* ~34 MYA, and a WGD event took place about 2─3 MYA. Throughout the evolutionary history of *F. carica*, chromosomes 2, 8, and 10 had experienced chromosome recombination, while chromosome 3 saw a fusion and fission. It is worth proposing that the chromosome 9 experienced both inversion and translocation, which facilitated the emergence of the *F. carica* as a new species. And the selections of *F. carica* for the genes of recombination chromosomal fragment are compatible with their goal of domestication. In addition, we found that the *F. carica* has the *FhAG2* gene, but there are structural deletions and positional jumps. This gene is thought to replace the one needed for female common type *F. carica* to be pollinated. Subsequently, we conducted genomic, transcriptomic, and metabolomic analysis to demonstrate significant differences in the expression of *CHS* among different varieties of *F. carica*. The *CHS* playing an important role in the anthocyanin metabolism pathway of *F. carica*. Moreover, the *CHS* gene of *F. carica* has a different evolutionary trend compared to other *Ficus* species. These high-quality genome assembly, transcriptomic, and metabolomic resources further enrich *F. carica* genomics and provide insights for studying the chromosomes evolution, sexual system, and color characteristics of *Ficus*.

## Introduction

*Ficus carica* L. (*Fig*), a member of the Moraceae family’s genus *Ficus*, is a heterozygous species (Mori et al., 2017). The reason it is termed “*fig*” is that the tiny flowers that are concealed in the hypanthium are not visible from the outside, only the pseudo-fruit formed by the receptacle. Generally accepted to have originated in Southwest Asia and the Middle East, *F. carica* are among the earliest known domesticated species, having been grown over 11,000 years (Kislev et al., 2006; Simsek et al., 2020). This species can tolerate extreme environmental conditions and poor soils (Vangelisti et al., 2019), and offer significant nutritional and health benefits (Vinson et al., 2005; Solomon et al., 2006; Veberic et al., 2008). *F. carica* has garnered a lot of attention lately as a promising functional food and drug candidate with high pharmacological activity because of their remarkable flavor and a variety of bioactivities (Purnamasari et al., 2019; Ayuso et al., 2022).

*F. carica* has significant therapeutic qualities, and research has looked into the possible use of common *fig* in the treatment of COVID-19 infections (Hamed et al., 2023). In addition, *F. carica* has a significant commercial value and is a major crop in the majority of Mediterranean nations as well as the US. The production of *F. carica* is anticipated to exceed one million tons year, the fruit’s consumption has increased globally and is predicted to continue growing in the years to come (Harzallah et al., 2016).

There are over 800 species in the genus *Ficus*, which is one of the largest genera in angiosperms. It was discovered that most *Ficus* species were diploid, having 13─14 chromosomes (2n=26, 2n=28). Among them, *Ficus microcarpa* (*F. microcarpa*) has 13 chromosomes with an assembled genome size of 436 Mb and is monoecious, while *Ficus hispida* (*F. hispida*) has 14 chromosomes and is dioecious (Zhang X. et al., 2020). *Ficus erecta* (*F. erecta*), a wild relative of common *F. carica*, has a genome size of 331.6 Mb and a Contig N50 of 1.9 Mb (Shirasawa et al., 2020). The first reported *F. carica* genome sequence is the Japanese cultivar, Horaishi, with a total assembled genome length of 248 Mb, Contig N50 of 4.5 Kb, and an estimated size of 356 Mb. The total length of the assembled genome is approximately 30% shorter than the estimated size (Mori et al., 2017). Later, the genome of another Italian *fig*, “Dottato,” was also published, with a total length of 333 Mb and a Contig N50 of 823 Kb. And 80% of the assembled genome was allocated to 13 chromosomes (Usai et al., 2020). As the most commercially significant species in the *Ficus* genus (Mawa et al., 2013), *F. carica* requires the assembly of a more comprehensive genome. In addition, chromosomes, which contain crucial genetic information for eukaryotes, have experienced a variety of intricate alterations during the course of the lengthy evolutionary engineering of organisms. Genome-wide duplication events are the first type of alterations in chromosome number, their importance in speciation and the development of new species cannot be overlooked (Ruprecht et al., 2017) and repeated rounds of WGD events can periodically boost plant genetic diversity (Mandakova et al., 2010). Chromosome chance events in unique contexts are the second category, wherein the number of individual chromosomes is either increased or decreased. The offspring inherit this alteration steadily and with retention. Chromosome rearrangement is one of the most interesting chromosomal occurrences. When DNA double strand breaks are being repaired, an unusual type of recombination called chromosome rearrangement takes place. Chromosome rearrangement consists of a variety of changes such as chromosome insertions, deletions or duplications, inversions, translocations, and transpositions. The term “chromosomal translocation” refers to the movement of chromosome segments from one chromosome to another, duplicate chromosome segments on distinct chromosomes are also thought to be the outcome of this process. Translocation and inversion are the two types of chromosomal recombination that can lead to secondary recombination of chromosomes and changes in chromosome structure (Schubert and Lysak, 2011), which can further modify the karyotype of the organism. These have the potential to alter chromosomal numbers, which could lead to the emergence of new species and the diversification of existing ones (Schubert and Lysak, 2011; Romanenko et al., 2019).

Caprifig, Smyrna, San Pedro, and common *fig* are the four types of *F. carica* that can be distinguished by their reproductive and pollination traits. *F. carica* trees are gynodioecious with two majors sex types: the caprifig and *fig* types. Though caprifigs are hermaphrodite plants with both male and female blooms, they solely function as male plants because they can only bear pollen and not edible fruit. However, female blooms continue to be essential for artificial feminization or wasp pollination of some *fig* species (Ikegami et al., 2013). More than half of *Ficus* plants display dioecy from a functional standpoint. Reportedly, the sexual orientation of *F. carica* is determined by the *RAN1* gene (Mori et al., 2017). However, studies have demonstrated that the *RAN1* gene does not exhibit clear gender or organ specificity in its expression. The *AGAMOUS* paralogous homologous gene *FhAG2* was shown to be the candidate gene accountable for male-specific gender identity in hispida (Zhang, X., et al., 2020). These investigations offer guidance and important data for the study of the genomes of *Ficus* plants, and they will be important for future investigations into the genes of dioecious and unisexual plants. Further, *F. carica* comes in a variety of colors, including yellow, green, red, purple. Researches have shown that the red peel of *F. carica* is mainly determined by the content of anthocyanins, while the yellow peel is mainly due to the high content of carotenoids, the green peel is mainly result from the high content of chlorophyll. *F. carica* peels contain four different types of anthocyanins: pelargonin-3-glucoside, cyanidin-3,5-diglucoside, cyanidin 3-glucoside, and cyanidin-3-rutinoside (Duenas et al., 2008; Treutter et al., 2010; Zhang H. et al., 2020). Previous researchers have examined the anthocyanin biosynthesis route. Essentially, 4-coumaric acid is produced by phenylalanine, and 4-coumaric acid CoA ligase (4CL) catalyzes the creation of 4-coumaric acid 4-coumaric CoA. 4-Enzymes involved in anthocyanin synthesis work with fumaric acid CoA and another precursor, malonyl CoA, to produce stable anthocyanins in the end (Castellarin et al., 2007; Czemmel et al., 2012; Zhang et al., 2014). The anthocyanidin biosynthesis pathways in plants have been extensively studied (Tanaka et al., 2008) and are associated with many genes and transcription factors. However, the ‘anthocyanin synthesis pathway’ related to the variations in the flesh color in the different varieties of *F. carica* has been rarely studied.

Currently, there is a vast and varied range of *F. carica* varieties, and scientific research on *F. carica* is continually growing. Genetic and breeding studies will have greater benefit from a more complete genome. Furthermore, no publications have been published on the transcriptome and secondary metabolome of different varieties, which is extremely important for *F. carica* genome mining and genetic improvement. In this study, Orphan *fig* was selected as the research material and third-generation long-segment nanopore sequencing was used to sequence the young and fresh leaves of Orphan *fig*. As a reference genome, the excellent Orphan genome was built. Joint analysis was performed using the acquired *F. carica* genome in conjunction with transcriptome and secondary metabolome analysis.

## Materials and methods

### Plant material and library construction

Utilizing improved Cetyltrimethyllammonium Bromide (CTAB) method was used to extract lengthy DNA segments weighing more than 500 ng from the tender leaves of Orphan (A212) (**Supplementary Figure 1**). Afterwards, the purified library was sequenced using a nanopore sequencer (Oxford Nanopore Technologies, Oxford, UK). After tender *fig* leaves were fixed in formaldehyde, the cells were lysed, and samples were taken out to assess the quality. Following biotin labeling, blunt-end ligation, chromatin digestion with restriction enzymes, DNA extraction, and purification, Hi-C samples were made and their DNA quality examined. A standard library was built once the quality test was passed. The NovaSeq platform (Illumina, San Diego, CA, USA) was used for the sequencing. Fastp (version 0.23.0) (Chen et al., 2018) with default parameters was used to filter adaptor contamination and low-quality reads in order to get clean sequencing data.

### Genome assembly and quality assessment

Nanopore-derived reads were corrected using NextDenovo (https://github.com/Nextomics/NextDenovo) and then used as input for SMARTdenovo assembly (Istace et al., 2017). After the initial assembly, polishing was repeated with NextPolish (Hu et al., 2020). The valid end reads obtained based on the Hi-C data were used to assist with genome assembly. Using 3D DNA pipeline (https://github.com/theaidenlab/3d-dna), the contigs were divided into subgroups and reassembled (Olga et al., 2017). In addition, a BUSCO (Benchmarking Universal Single-Copy Orthologs) (Seppey et al., 2019) assessment of the genome was performed to evaluate the entirety of the assembled genome.

### Genome and TF annotation

Repeat sequences in the *F. carica* genome were identified based on self-BLAST (https://github.com/Dfam-consortium/RepeatModeler) using the RepeatModeler (version 1.0.10) (https://github.com/Dfam-consortium/RepeatModeler) (Flynn et al., 2020). RepeatMasker (version 4.0.7) (http://www.repeatmasker.org) cross-matching was used to search further for known repeats. A pipeline integrating *de novo* gene prediction and RNA-seq gene model was used to predict the protein-encoding genes. For *de novo* gene prediction, Augustus (version 3.0.2) (Stanke et al., 2006) and SNAP (https://github.com/KorfLab/SNAP) were run with default parameters. For RNA-seq-based prediction, RNA-seq reads were screened from the pooled tissue samples to eliminate the adapters and trimmed to remove low-quality bases. The processed reads were then aligned with the reference genome.

### Construction of evolutionary tree and estimation of evolution rate

Homologous gene families were identified in the genomes of *Ficus carica*, *Ficus hispida, Ficus microcarpa, Morus alba, Cannabis sativa, Ziziphus jujuba, Arabidopsis thaliana, Carica papaya, Citrus sinensis, Manihot esculenta, Vitis vinifera*. To construct protein gene sets of multiple species, the encoded protein sequences were obtained from the genomic data of the species mentioned. OrthoFinder (version 2.2.6) (Emms and Kelly, 2019) was employed to cluster the selected protein sequences and identify orthologous genes by screening for genes with low-copy numbers. Single-copy, homologous genes were identified from the collection and used to construct an evolutionary tree (Price et al., 2010). The evolutionary tree was converted to a time tree using r8s Calibrate Time of the Timetree database (http://www.timetree.org/) (Kumar et al., 2017). CAFÉ (version 4.1) (De et al., 2006) was employed to analyze the expansion and contraction of gene families based on the chronogram of the 11 species.

### Collinearity and Ks analysis

MCScanX (Wang et al., 2012) set to default parameters was used to identify the collinear genes, and proteins were used to screen the genomes of species to obtain the best matching pair. Each aligned block represented an orthologous pair derived from a common ancestor. Ks (synonymous substitutions per synonymous site) values for the homologs within the collinear block were determined using PAML (version 4.5) (Yang, 2007). The median Ks value was regarded as the representative of the collinear block. The hypothetical whole-genome replication and the putative whole-genome duplication (WGD) events in *F. carica* were identified by plotting the values of all gene pairs. The formula t = Ks/2r representing the neutral substitution rate was used to estimate the replication and differentiation times between *F. carica* and other species. The neutral substitution rate used in this study was 8.12 × 10^9^. Calculation of ka/ks was performed using the KaKs_Calculator (http://evolution.genomics.org.cn/software.htm) (Zhang et al., 2006) software. when Ka is equal to Ks (ka/ks = 1), indicating a neutral mutation; when Ka is less than Ks (ka/ks < 1), it indicates a negative (purification) selection; when Ka exceeds Ks (ka/ks > 1), it indicates a positive (diversification) selection.

### Analysis of the secondary metabolome

The samples from four varieties of *F. carica*, F1: “Orphan,” F2: “Balaonai,” F3: “Violette Solise,” and F4: “Bpjihon,” each with a different fruit flesh color were collected. The secondary metabolites from each variety were extracted in triplicate. Ultra-high-performance liquid chromatography was the primary analytical system used, and the data obtained was scrutinized by the Analyst 1.6.3 software. Metabolites with a fold change ≥ 2 or ≤ 0.5, P-value < 0.05, and item variable importance ≥ 1 were considered statistically significant. Using the KEGG compound database, the metabolites identified were annotated to the KEGG pathway database (Kanehisa et al., 2017).

### Transcriptome sequencing

The fruits of F1, F2, F3, and F4 were harvested in three biological repetitions and immediately frozen in liquid N_2_. RNA seq analysis included RNA isolation, library construction, and sequencing for gene prediction. Raw data was trimmed to eliminate the adapters and improve the quality. The reads < 100 bp long were discarded. TopHat2 (version 2.0.4) (Kim et al., 2013) was used for mapping the clean reads to the genome under default parameters. The transcript was assembled using Cufflinks (version 2.2.1) (Trapnell et al., 2012). The gene expression levels were measured using transcriptional fragments plotted from Cufflinks per kilobase bases per million fragments, and the differentially expressed genes (DEGs) were determined with DEseq2 (Varet et al., 2016). The expression data for different breeds were centralized, normalized, and then clustered using K-means to analyze the differential gene expression patterns. False discovery rates were used for adjusting P value. The genes with statistically significantly different expression levels, i.e., |log_2_(fold change)| ≥ 1 and adjusted P values < 0.05, were identified as DEGs and annotated using the GO enrichment and KEGG pathways.

### Functional gene analysis

Ten gene families in the *F. carica* genome, including *PAL*, *C4H*, *4CL*, *CHS*, *CHI*, *F3H*, *F3`H*, *DFR*, *ANS*, and *UFGT*, were detected from the HMM domain model and using BLASTP (version 2.2.3.1) by studying the pathways involved in the regulation of fruit color, especially the “anthocyanin synthesis pathway.” *Fig* genome was screened for identification by employing the HMMER (version 3.0) software. Then, conserved domains were confirmed in all the protein sequences, while those with incomplete domains were excluded. The Pfam database (El-Gebali et al., 2019) was used to predict the domains of these protein homologs, and the genes that encoded proteins with identical domains were considered homologs.

## Results

### Sequencing and assembly of the genome

Using *K*-mer analysis, the size of the *F. carica* genome was estimated to be 356 Mb. In total, 3,301,024 reads amounting to 42 Gb were generated, with a sequencing depth of ~ 100× and an average read length of 12.78 Kb. A high-throughput chromosome concept capture (Hi-C) library of the genome of “Orphan” was constructed to enhance the quality of the assembly and mount the contigs on chromosomes, which resulted in 64.26 GB of Hi-C paired ends at 140× (**Supplementary Table 1**).

After genome assembly, polishing, and elimination of redundancy, the final size of the genome was 373.72 Mb, and that of the contig N50 was 9.78 Mb (**Supplementary Table 2**). The Hi-C heatmap was first examined, and a diagonal pattern of high link frequencies was observed in the individual pseudochromosomes, indicating increased interactions between adjacent regions (**Supplementary Figure 2**). Duplicate deletions, classification, and quality assessment were performed on Hi-C-Pro, and only the mapped, valid reads were used for Hi-C. As a result, a sequence 366.34 Mb in length was allocated to 13 chromosomes, accounting for 98.02% of the total length, with the number of corresponding contig cut bins obtained being 3,906 (**Supplementary Table 3**). The completeness and accuracy of the assembled genome were assessed using BUSCO, and over 96.2% of the BUSCO-derived assessments were located in the assembled genome (**Supplementary Figure 3**, **Supplementary Table 4**) (**Table 1**).

### Genome annotation

The *de novo* predicted genomic data were collected from the young leaves of *F. carica*, and the transcriptome data from the four samples of *F. carica*. Comparison between *F. carica* and *F. microcarpa* and *F. hispid*.

Collinearity analysis of *a*, the results show that *F. microcarpa* and *F. hispida* possessed 29,402 and 27,210 genes, respectively. In total, 29,783 protein-coding genes were identified within the *F. carica* genome, of which 29,039 were mapped to specific chromosomal loci, accounting for 97.5% of the total genome, with an average gene length of 3,111 bp, and a coding sequence length of 32,006,639 bp penetrance (exon). The mean GC content was 34.13%, higher than that reported in a previous study (33.38%). A total of 812,147 repeat sequences were identified in the assembled genome, accounting for 47.92% of the total genome, which too was higher than the value of 20.9% reported previously. Of these repeats, LTRs accounted for 10.11% (most abundant) and transposons for 3.26% (**Supplementary Table 5**) (**Table 1**).

**Table 1:** Statistics for assembly and annotation of the *F. carica* genome.

Sequencing			
Sequencing platform	NovaSeq6000	Nanopore	Hi-C
Cleaned data (Gb)	26	42	62
Genome sequencing depth (×)	75	100	140
**Assembly**	**Orphan**	**Dottato (** Usai et al., 2020)	**Horaishi (** Mori et al., 2017)
Assembled genome size (Mb)	373,718,651	333,400,567	247,090,738
Sequence assigned to chromosomes (Mb)	366,336,389	266,522,563	–
Number of chromosomes	13	13	–
Max Contig (bp)	22,062,110	5,010,936	1,764,766
Min Contig (bp)	37,148	20,012	479
Contig N50 (bp)	9,781,938	823,517	166,092
BUSCO completeness (%)	96.2	93.3	90.5
Annotation			
GC content (%)	34.2		
Number of protein-coding genes	29,783		
Average gene length (bp)	3,111		
Average exon number per gene	5.2		

### Evolution, genome-wide replication, and species collinearity in *F. carica*

**Figure 1:**
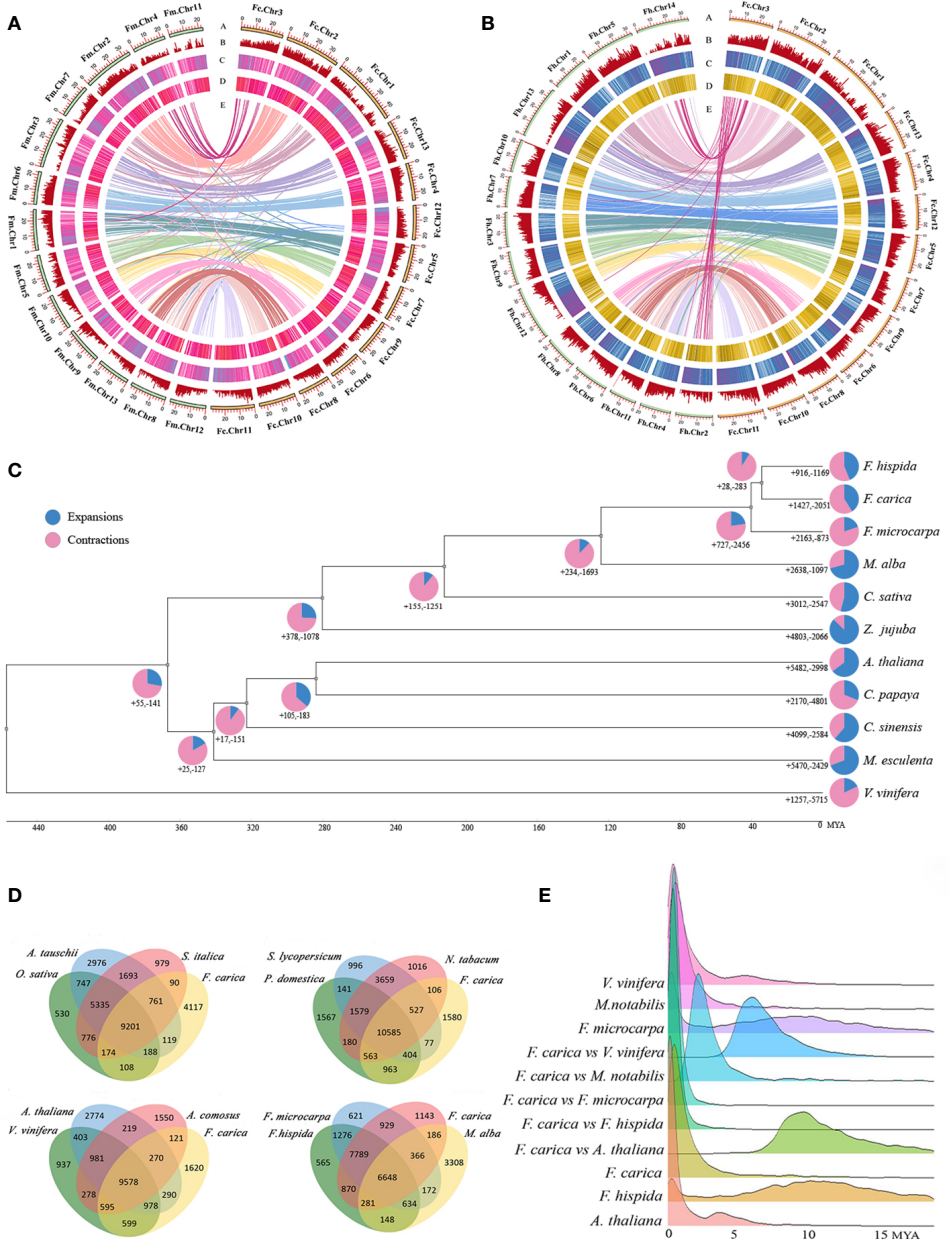
Phylogenomics and genomic evolution. **(A)** Genomic features of *Ficus carica* and *Ficus microcarpa*. **(B)** Genomic features of *Ficus carica* and *Ficus hispida*. The Circos plot of the multidimensional topography depicted from the outermost to innermost, **(A–E)** chromosome karyotypes, gene density, LTR Tes, DNA Tes, and synteny between the two genomes. **(C)** Phylogenetic tree, divergence times, and expansion and contraction of gene families in 10 species and *Ficus carica*. Pie charts indicate the proportion of gene families that underwent expansion and contraction. **(D)** Veen diagram showing the shared and unique gene families among *Ficus. carica*, *Aegilops tauschii*, *0ryza sativa*, *Setaria italica*, *Solanum lycopersicum*, *Prunus domestica*, *Nicotiana tabacum*, *Arabidopsis thalian*a. *Vitis vinifera*, *Ananas comosus*, *Ficus microcarpa*, *Ficus hispida*, *Morus alba*. **(E)** *Ks* distribution of *Vitis vinifera*, *Morus notabilis*, *Ficus microcarpa*, *Ficus carica*, *Ficus hispida*, and *Arabidopsis thaliana*, between *Ficus carica* and *Vitis vinifera, Morus notabilis*, *Ficus microcarpa*, *Ficus hispida*, and *Arabidopsis thaliana*.

Collinearity analysis of the *F. carica*, *F. hispida*, and *F. microcarpa* genomes suggested that a total of 9,926 single-copy, homologous gene pairs between *F. carica* and *F. microcarpa*; and 9,606 between *F. carica* and *F. hispida* were identified. Analysis of the homologous genes demonstrated a close evolutionary relationship between these three species. *F. hispida* had a substantial congruity with *F. carica*; chromosome 14 and chromosome 2 of *F. hispida* had a remarkable conformity with chromosome 1 of *F. carica*. In addition, 19,967 and 19,825 collinear genes were identified Between *F. carica* and *F. hispida*, *F. microcarpa*, respectively, indicating that 67% and 66% of the *F. carica* genome was collinear with those of the respective species. These results further indicated that the ancestors of *F. carica* may have undergone chromosomal fusions or divergences (**Figure 1A**, **B**).

A phylogenetic tree was constructed using r8s, which showed that *F. carica* was closely related to *Z. jujuba* (Rhamnaceae), *C. sativa* and *M. alba* (Moraceae). The divergence of *F. carica* and *F. hispida* from *F. microcarpa* occurred approximately 40 million years ago. *F. carica* then diverged from *F. hispida* about 34 million years ago. The expansion and contraction of gene families are crucial characteristic features of species selective evolution. Analysis showed that *F. carica*, *F. microcarpa*, and *F. hispida* acquired new genes and gene families during evolution. However, during the evolution of each species, independent gene families were acquired and lost to varying degrees. *F. hispida* and *F. carica* underwent expansion of 727 gene families and contraction of 2,456 gene families after differentiation from *F. microcarpa*. While in *F. carica*, 1,427 gene families were expanded, and 2,051 were contracted. In the *F. hispida* evolution node, 916 gene families were expanded, and 1,169 were contracted (**Figure 1C**).

Comparative analyses of the gene families revealed 9,201 gene families common to *O. sativa*, *S. italica*, and *A. tauschii*, 10,535 to *S. lycopersicum*, *P. domestica*, and *N. tabacum*, 9,578 to *V. vinifera*, *A. thaliana*, and *A. comosus* and 6,648 to *F. microcarpa, F. hispida*, and *M. alba*. Compared with *F. microcarpa* and *F. hispida*, 6,199 gene families were unique to *F. carica* (**Figure 1D**, **Supplementary Table 6**). GO analysis of these families revealed that ‘transporter activity’ and ‘transcription regulator activity’ were significantly enriched (**Supplementary Figure 4**). The protein sequences of *F. carica, F. hispida*, *F. microcarpa*, and *A. thaliana*, were compared using Blastp. The number of homologous genes identified was 2,943 between *F. carica* and *F. hispida*; 2,079 between *F. carica* and *F. microcarpa*; and 4,295 between *F. carica* and *A. thaliana*. There were 8,142 homologous genes in *F. carica*, 9,849 in *F. microcarpa*, and 6,272 in *F. hispida*. Based on the comparisons of the homologous genes, the time point when the *F. carica* underwent genome-wide replication was ascertained, and the synonymous substitution rate (KS) was calculated. The KS values of the homologous gene pairs between *F. hispida, F. microcarpa*, and *F. carica* were calculated to judge the time point of differentiation. The results obtained showed that *F. microcarpa* differentiated earlier than *F. carica* and *F. hispida*, while *F. hispida* and *F. carica* were closely related and differentiated in 5 million years, which was consistent with the results of the evolutionary time tree. Each peak of KS in the genome represents a genome-wide replication (WGD) event. However, the peak close to the vertical axis on the left results from repeats in the genome and is not an actual KS peak. Therefore, as shown in **Figure 1E**, *F. carica* must have undergone WGD events during evolution 2─3 million years.

### Chromosomal evolutionary analysis of *F. carica* with *F. microcarpa* and *F. hispida*

**Figure 2:**
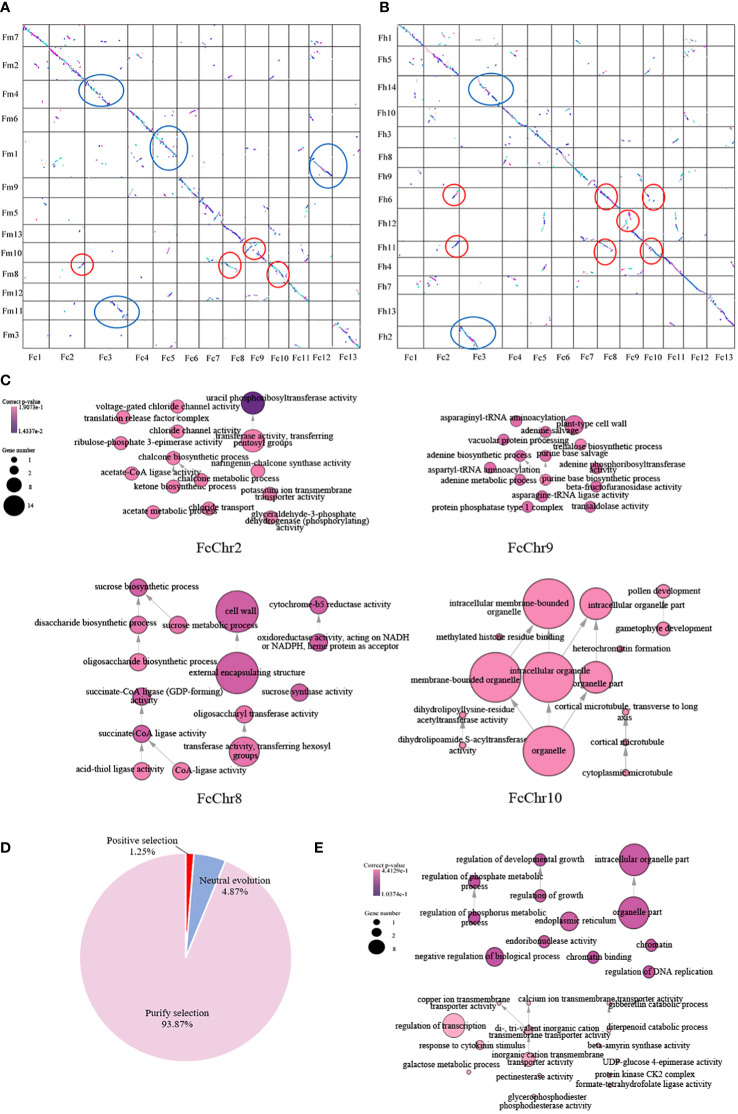
Chromosomal evolutionary analysis of F. carica with F. microcarpa and F. hispida. **(A)** Chromosomal collinearity of *Ficus carica* and *Ficus microcarpa*. **(B)** Chromosomal collinearity of *Ficus carica* and *Ficus hispida*. Chromosome fusion and fission are circled in blue ellipses, while chromosome recombination is outlined in red ellipses. Chromosome inversion and translocation are circled in red. **(C)** Functional enrichment of genes involved in recombination of *Ficus carica* chromosomes 2, 8, 9, and 10. **(D)** Analysis of selection pressure for *F carica* and *F hispida*, including positive selection (ka/ks > 1), neutral evolution (ka/ks = 1), purify selection (ka/ks = 1). **(E)** Functional enrichment of positive selection genes in *Ficus carica*.

Strong correspondences have been observed between the chromosomes of *F. carica*, *F. microcarpa*, and *F. hispida*. Several instances of chromosome fusion and breakage between the chromosomes of *F. carica* and the two *Ficus* species were discovered by further collinearity analysis. As *F. carica* differentiated from *F. microcarpa*, chromosomes 4 and 11 of *F. microcarpa* joined together to become chromosome 3 of *F. carica*. Moreover, the chromosome 1 of *F. microcarpa* splits to generate chromosomes 5 and 12 in the *F. carica*. In addition, chromosome fusion and breakage events between *F. carica* chromosome 3 and *F. hispida* chromosomes 2 and 14 occurred throughout the process of *F. carica* and *F. hispida* development. Chromosome variation has long been known to encourage the emergence of new species and the diversification of existing ones. In the analysis of the covariation between *F. carica* and two *Ficus* species, it was found that after *F. carica* diverged from *F. microcarpa*, the inversion of the 3.59 Mb fragment at the end of *F. carica* chromosome 2, the chromosome duplication occurred on the 12.04 MB fragment at the anterior end of chromosome 8 as well as on the 10.77 Mb fragment at the anterior end of chromosome 10, and furthermore the 12.44 Mb fragment at the anterior end of *F. carica* chromosome 9 also had a chromosomal translocation. In addition, chromosomal translocations and inversions occurred on the 12.44 Mb segment of the anterior end of *F. carica* chromosome 9. Coincidentally, after *F. carica* diverged from *F. hispida*, *F. carica* chromosome 2 was also inverted and duplications occurred on chromosomes 8 and 10, and similarly, chromosomal translocations and inversions occurred on the anterior end of *F. carica* chromosome 9 (**Figure 2A**, **B**). *F. carica* has a chromosome number of 13, whereas *F. hispida* has 14 chromosomes. This difference in chromosomal number can be attributed to the chromosomal recombination events previously mentioned.

GO enrichment was performed for genes with mutated segments on *F. carica* chromosome 2, chromosome 8 and 9, and chromosome 10. It was found that these genes were mainly enriched in ‘chalcone metabolic process’, ‘chalcone biosynthetic process’, and ‘chalcone synthase’. Chalcone synthase is the first enzyme in the synthesis pathway of plant flavonoids, which is not only closely related to plant fertility, but also plays an important role in plant resistance to pathogens. In addition to this, they were also enriched in ‘gametophyte development’, ‘sucrose synthase activity’, ‘sucrose biosynthetic process’, ‘sucrose metabolic process’ (**Figure 2C**). Compared to the two *Ficus* species, it is worth suggesting that *F. carica* trees are shorter in height and have edible fruits. The objective of their domestication is also reflected in the choice of *F. carica* for gene selection. This conclusion can be further demonstrated by looking at the selection pressure analysis of *F. carica* and *F. hispida.* Positive selection (**Figure 2D**) is mostly carried out by *F. carica* during the evolutionary process on genes associated with processes like ‘negative regulation of developmental growth’, ‘negative regulation of growth’, and ‘regulation of cellular biological process’ (**Figure 2E**).

### Analysis of gene related to sex determination in *F. carica*

**Figure 3:**
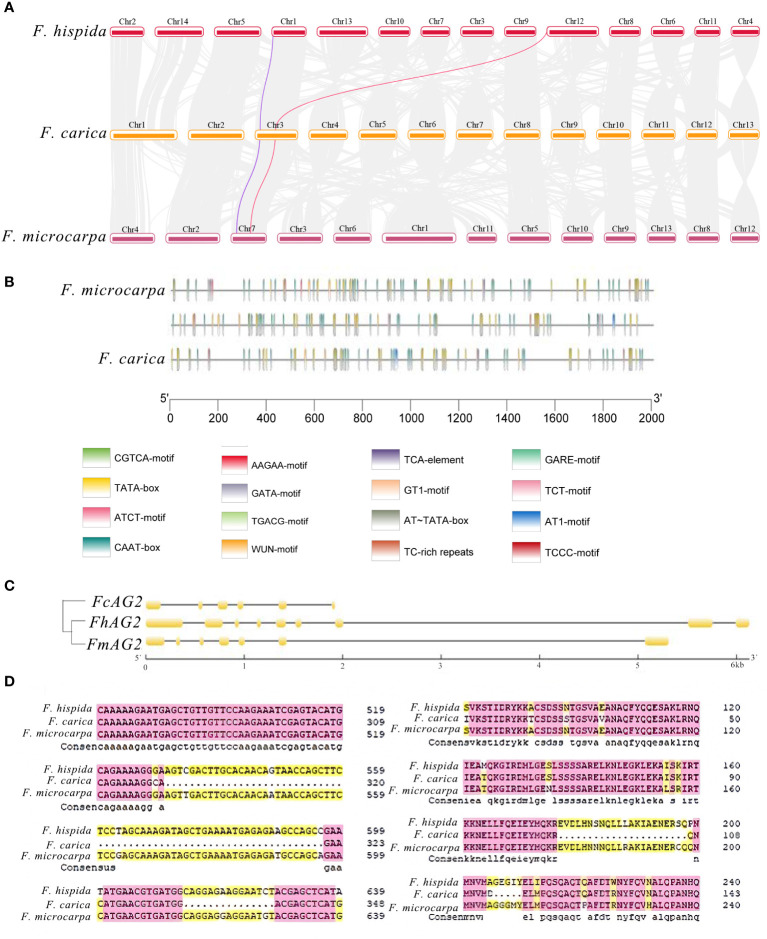
Analysis of Genes Related to Sex Determination in *F carica*. **(A)** Collinearity analysis of the male and female sex-determining genes of *F carica* and *F hispida*, *F microcarpa* (the purple line indicates *RAN1* and the red line, *FhAG2*). **(B)** CIS original analysis of the *F carica*, *F hispida*, and *F microcarpa* promoters. **(C)** Structural analysis and the evolutionary tree of AG genes in the *F carica*, *F hispida*, and *F microcarpa*. **(D)** Alignment of the *F carica*, *F hispida*, and *F microcarpa* protein sequences.

Both *F. carica* and *F. hispida* are dioecious *Ficus* species. In the dioecious *F. carica*, the protein-coding gene *Fh.AG2* unique to males was discovered. Sequence alignment revealed similar genes on chromosome 3 of *F. carica* and in the hermaphrodite *F. microcarpa*. The *Fh.AG2* gene jumped on the chromosomes of *F. carica* and *F. microcarpa*, according to a comparison of their positions on the chromosomes with *F. hispida* (**Figure 3A**). Alignment of gene protein domains showed two obvious deletions in the protein domain of *F. carica* compared with that of *F. hispida* and *F. microcarpa*. Furthermore, there were many protein-coding gene regions that *F. carica* and *F. hispida*, *F. microcarpa* shared (**Supplementary Figure 5**). The cis-acting elements in the promoters of this gene family were discovered by comparing the first 2000 bp of the AG gene promoters of these three *Ficus* plants. Additionally, it was discovered that the 400-2000 bp of *F. microcarpa* and the first 1600 bp of *F. hispida* shared comparable promoter structure. Compared with the other species of italy Ficus, the promoter in *F. hispida* was mainly a CAAT-box, and that in *F. carica* was mainly a CAAT-box and a TATA-box. The main functions of these promoters and the common *cis*-acting elements of the promoters and enhancers are transcription initiation around the core promoter element at -30, respectively (**Figure 3B**).

The AG gene structure comparison between *F. carica* and *F. hispida* and *F. microcarpa* revealed that there were clear deletions in the CDS structural domain of the *F. carica* genes. The two *Ficus* varieties and *F. carica* evolved relatively separately, according to the evolutionary tree built from the CDS sequences (**Figure 3C**). From the perspective of protein structure, the relationship between *F. hispida*, *F. carica*, and other dioecious plants was much stronger than that with *F. microcarpa* (**Figure 3D**). During evolution, the genes involved in sex determination in *F. hispida* and *F. carica* underwent a specific differentiation. The Ka/Ks ratio of *F. microcarpa* and *F. carica* was 1.04, indicating that the gene was affected by positive selection in these species.

### Transcriptome sequencing, clustering, and functional enrichment

**Figure 4:**
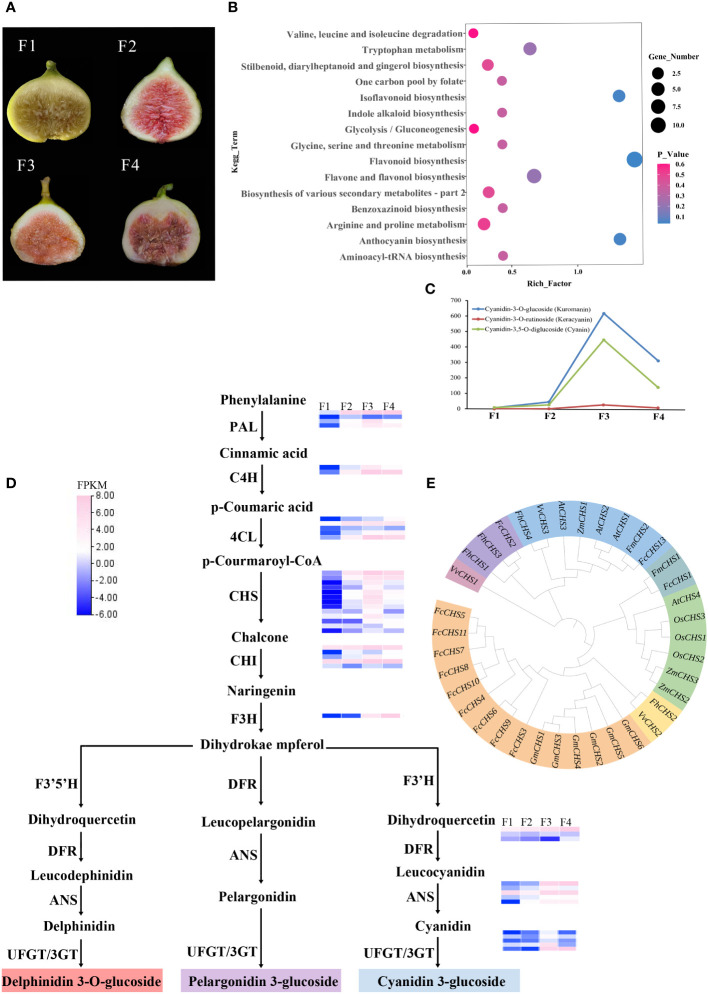
Transcriptome metabolism analysis of four different colors of *F carica*. **(A)** The varieties of *F carica* with different colored fruits. F1: “Orphan,” F2: “Balaonai,” F3: “Violette Solise,” F4: “Bpjihon.” **(B)** Pathway enrichment analysis of the differentially-accumulated metabolites in F1 vs. F3. **(C)** Changes in the levels of anthocyanins in the four different varieties of *F carica*. **(D)** Flavonoids biosynthesis pathway and gene expression in the four varieties of *F carica* with different colored fruits. Gene expression levels (log2-based FPKM) in different varieties are represented by color grading. **(E)** Phylogenetic tree of the *CHS* gene family in various species.

Transcriptome sequencing of four differently colored *F. carica* fruits was performed (**Figure 4A**). After filtering raw data, checking error rates, and determining the GC content, 45.98-63.77 million high-quality, 150 bp base raw data were obtained. The clean reads were then mapped to the genome, and more than 90% could be successfully aligned (**Supplementary Table 7**). From the transcriptomes of the four groups of samples, 2,582 DEGs (1,797 up-regulated and 785 down-regulated) were identified between F1 and F2, 3,445 DEGs (2,272 up-regulated and 1,173 down-regulated) between F1 and F3, and 3,062 DEGs (1,924 up-regulated and 1,138 down-regulated) between F1 and F4 (**Supplementary Figure 6**). In total, 980 DEGs and 47 differentially expressed TFs were also identified to be common to the four sets of data (**Supplementary Table 8**, **Supplementary Figure 7B, C**).

The expression patterns of the genes identified in the three stages of fruits were divided into ten subfamilies (**Supplementary Figure 8**). GO enrichment showed that F1 and F2 were significantly enriched in ‘secondary metabolic processes’, ‘chloroplast thylakoid’, ‘plasma membrane fraction’, ‘plastid thylakoid’, ‘thylakoid’, ‘ADP binding’, ‘heme binding’, ‘monooxygenase activity’, ‘oxoquinene cyclase activity’, ‘tetrapyrrole binding,’ and ‘UDP glucosyltransferase activity’ (**Supplementary Figure 9**). For F1 and F3, ‘bacterial defence responses’, ‘secondary metabolic processes’, ‘secondary metabolite biosynthesis’, ‘chloroplast thylakoid’, ‘components of the plasma membrane’, ‘parts of the plasma membrane’, ‘hydrolase activity acting on glycosidic bonds’, ‘hydrolase activity’, ‘hydrolysis of oxyglycosyl compounds’, ‘monooxygenase activity,’ and ‘tetrapyrrole binding’ were significantly enriched (**Supplementary Figure 10**). For F1 and F4, “cell death,” ‘symbiotic-induced host-programmed cell death’, ‘response to auxin’, ‘secondary metabolic process’, ‘secondary metabolite biosynthesis process’, ‘chloroplast thylakoids’, ‘neutral components of plasma membrane’, ‘parts of plasma membrane’, ‘thylakoids’, ‘ADP binding’, ‘heme binding’, ‘oxygenase reductase activity,’ and ‘tetrapyrrole binding’ were significantly enriched (**Supplementary Figure 11**).

Differential gene expression analysis was also performed using KEGG enrichment. Significant enrichment was found in ‘plant-pathogen interaction’, ‘plant hormone signal transduction’, ‘phenylpropanoid biosynthesis,’ and ‘secondary metabolite biosynthesis’ between F1 and F2 (**Supplementary Figure 12**), ‘plant-pathogen interaction’, ‘phenylpropane biosynthesis,’ and ‘biosynthesis of secondary metabolites’ between F1 and F3 (**Supplementary Figure 13**), and ‘plant-pathogen interaction’, ‘phenylpropanoid biosynthesis’, ‘flavonoid biosynthesis,’ and ‘biosynthesis of secondary metabolites’ between F1 and F4 (**Supplementary Figure 14**).

### Differences in the secondary metabolites of *F. carica* varieties

Sequencing the transcriptome of the four varieties of *F. carica* suggested remarkable differences in the secondary metabolic processes of the species. The four differently colored fruits of *F. carica* were collected to understand the underlying mechanisms. The metabolic analysis conducted in this study identified 348 secondary metabolites, which included terpenes, flavonoids, and phenolic acids. Principal component analysis was performed on the data obtained from the gas chromatography-mass spectrometry to compare the differences in the metabolites of the ripe fruits of the four varieties of *F. carica*. Principal component analysis could easily distinguish between F1, F2, F3, and F4. Using the scoring graphs of PC1 and PC2, the compositions of the metabolites in the four samples could be distinguished. PC1 and PC2 were separated among the four groups of samples. PC1 and PC2 explained 35.8% and 25.08% of the total variance, respectively (**Supplementary Figure 7A**).

### Differences in the levels of metabolites that affect fruit color in the four varieties of *F. carica*

Color is one of the critical characteristics considered for research related to improving horticultural plants. Fruit color is mainly affected by anthocyanins or carotenoids. The levels and types of metabolites may play a crucial role in determining the color of fruits in *F. carica*. The four fruit samples showed variations in the contents of terpenes, phenolic acids, and flavonoids. A total of 128, 129, and 111 differentially-accumulated secondary metabolites were identified between F1 and F2, F1 and F4, and F1 and F3, respectively. These included 51 metabolites that increased, and 76 that decreased between F1 and F2; 62 increased, and 67 decreased between F1 and F3; and 67 increased. and 44 decreased between F1 and F4. KEGG database was used to annotate the differentially-accumulated metabolites, and the results obtained indicated that the pathways that were mainly enriched included: ‘flavonoid biosynthesis’, ‘anthocyanin biosynthesis’, ‘isoflavone biosynthesis’, ‘phenylalanine biosynthesis,’ and ‘isoflavone biosynthesis’ (**Figure 4B**, **Supplementary Figure 7D**).

### Flavonoid biosynthesis pathway in the mature fruits of *F. carica*

Anthocyanins are flavonoids, water-soluble pigments that occur widely in plants and confer them with red, blue, and purple colors. A total of 94 flavonoids were detected through metabolomic analysis, of which anthocyanins were identified to be most closely associated with color. Three anthocyanins that were detected at significantly different levels in the assay were: cyanidin-3-O-glucoside (kuromanin), cyanidin-3-O-rutinoside (keracyanin), and cyanidin-3,5-O-diglucoside (cyanin), which were all up-regulated compared with those in F1. Amongst these, cyanidin-3-O-glucoside was 600-fold higher in F3 than in F1, while cyanidin-3,5-O-diglucoside was 400-fold higher (**Figure 4C**).

Anthocyanins are mainly synthesised through the anthocyanin pathway, providing abundant natural pigments for different tissues and organs of plants. They are generally synthesized through the phenylalanine pathway and play a role in producing various derivatives through different metabolic pathways. In anthocyanin pathway, ten crucial gene families were identified, most located on chromosome 10, chromosome 12, and chromosome 13. The results of the analysis demonstrated that in the four varied colored *F. carica* fruits, the expression levels of *4CL*, *CHS*, *CHI*, *F3H*, *ANS*, and *UFGT* increased and were compatible with the rising anthocyanin content. In particular, there are notable variations in the expression levels of *CHS* and *UFGT* among different varieties of *F. carica*. Subsequently, we discovered 31 family members through *CHS* gene family analysis. Most of these genes were linked and localized on chromosome 10 of *F. carica*. Additionally, tandem repeat sequences were found, which aid in the *CHS* gene family’s proliferation in *F. carica* (**Figure 4D**). A phylogenetic tree was also constructed based on the CDS sequences of *CHS* to determine the evolution of the *CHS* family in *F. carica*. It was discovered that *FcCHS3*, *FcCHS4*, *FcCHS5*, *FcCHS6*, *FcCHS7*, *FcCHS8*, *FcCHS9*, *FcCHS10* and *FcCHS11* are members of a subfamily. Notably, the *CHS* genes of *figs* show a distinct evolutionary tendency in comparison to other *Ficus* species (**Figure 4E**).

## Discussion

*F. carica* is one of the first species to be domesticated, have significant economic and utilitarian importance, and is widely cultivated throughout Southwest Asia and the Middle East. Nonetheless, *F. carica* genetic research has been impeded by the absence of greater genome availability. We present a high-quality genome of “Orphan” with a contig N50 of 9.78 Mb and 366.34 Mb (98.02%) allocated to 13 chromosomes, which is valuable for understanding the genetics and evolutionary relationship, providing genomic resources and new insights into the breeding of *F. carica*. The integrity of the genome of “Orphan” as a reference was higher than that of *F. microcarpa* (contig N50 of 908kb), *F. hispida* (contig N50 of 492kb) (Zhang X. et al., 2020), and the previously reported genome of *F. carica*, 248 Mb size (contig N50 of 4.5 Kb) (Mori et al., 2017). In this study, nanopore sequencing (Belser et al., 2018) and high-throughput chromosome conformation capture (Jiao et al., 2017) were used for the assembly of genomes with a high quality. The complete sequence of the *F. carica* genome serves as a significant resource for future studies regarding the evolution and molecular breeding in *Ficus*.

In the first type of chromosomal number change mechanism, whole gene replication events are a common and significant chromosomal event that are necessary for the formation of new species or distinct phenotypes during evolution (Otto, 2007). The estimated divergence time between *Ficus* and *Morus* was ~120 MYA, and the differentiation time of *F. carica* and banyan *F. hispida* was ~34 MYA. A WGD event occurred roughly 2─3 MYA after *F. carica* and *Ficus* separated, according to Ks analysis of the *F. carica* genome. Replication time gives redundant alleles unique or specialized activities, which may lead to the development of new regulatory mechanisms through genomic rearrangements. Chromosome fusion and breakage play equally important roles in the evolution of species. Chromosome number and ploidy will increase with biological evolution through whole genome duplication, polyploidization, and other processes. In addition, the genome may undergo diploidization to produce a small number of diploids, which will contribute to a decrease in chromosome number and ploidy. Related mechanisms include chromosome fusion and chromosome breakage (Mandakova et al., 2010; Soltis et al., 2016). Throughout their evolutionary history, *F. carica* have experienced chromosome fusion and fission with both *F. microcarpa* and *F. hispida*, including chromosome fusion and fission of chromosome 3 of *F. carica* with chromosomes 4 and 11 of *F. microcarpa*, and likewise in *F. carica* chromosome 3, which has also undergone chromosome fusion and fission with chromosomes 2 and 14 of *F. hispida*. It can be inferred from this that the chromosome 3 of *F. carica* is crucial for separating it from *Ficus* and creating a distinct species altogether.

An accidental event in a certain environment typically causes an increase or decrease in the total number of chromosomes in an organism during the course of its long-term evolution. This kind of chromosomal number alteration is referred to as the second kind. Progeny inherit a steady transmission of this change. Chromosomal recombination is the most interesting of these chromosomal events. Chromosome insertion, deletion, replication, inversion, translocation, and transposition are among the several modifications that can occur during chromosomal recombination. A chromosome can move from one chromosome to another, a process known as chromosomal translocation. Repeated regions on distinct chromosomes can also be attributed to chromosomal translocation. Chromosomes that experience translocation and inversion are likely to experience secondary recombination, and their structural changes will also impact the karyotype of the organism. All of these modifications have the potential to alter chromosomal numbers, which will promote the diversification and development of new species (Schubert and Lysak, 2011; Romanenko et al., 2019). Our research revealed that *F. carica* have experienced multiple chromosomal rearrangements across their evolutionary history, such as the inversion of chromosome 2 and the duplication of chromosomes 8 and 10. It’s also important to note that the chromosome 9 of *F. carica* underwent both translocation and inversion. The ‘chalcone metabolic process’ and ‘chalcone biosynthetic process’ are the primary areas of enrichment for the functions of genes that undergo chromosomal recombination in *F. carica*. Chalcone Synthase (CHS) is the first enzyme in the pathway leading to the synthesis of plant flavonoids, which are not only extremely associated with plant fertility but also significantly impact plant resistance to pathogen infestation. The primary location of flavonoid synthesis in pollen is the chorioallantoic layer. From there, the flavonoids are transferred to the cyst cavity and ultimately to the pollen grain’s outer wall, where they play a significant role. Thus, flavonoids are crucial for the development of pollen grains. Research has shown that the examination of the flavonoid content in anthers will help to verify that the development of male sterility in *CHS-A* transgenic plants may be caused by the transcription of CHS in anthers. Male sterility was also observed in the transgenic plants, which were successfully genetically modified to modify the color of the flowers when the positive *CHsA* gene was introduced into Petunia (Shao and Xiao, 1996). While *F. carica* are dioecious plants, *F. microcarpa* is monoecious. The sex-specific characteristics of *F. carica* are most likely the result of chromosomal recombination events that occurred after *F. carica* separated from *F. microcarpa.*

Furthermore, the chromosomal reorganization gene functions also associated with ‘gametophyte development’, ‘sucrose synthase activity’, ‘sucrose biosynthetic process’, and ‘sucrose metabolic process’. It is worth mentioning that *F. carica* fruit trees produce edible fruits and are shorter in height than the other two *Ficus* species. The selection of *F. carica* for these genes associated with development and growth as well as sugar synthesis is consistent with the goal of their domestication. The examination of selection forces on *F. carica* and *F. hispida* provides additional evidence for the argument. *F. carica* have evolved primarily in response to ‘negative regulation of growth’, ‘negative regulation of developmental growth’, ‘reaction to cytokinin stimulus’, and ‘regulation of cellular biosynthetic process’ genes with associated functions being positively selected. It is evident that the typical fruit characteristics of *F. carica* compared to other *Ficus* species can be attributed to the WGD event and Chromosomal recombinations. The genomic information of *F. carica* may facilitate the analysis of the evolutionary process undergone by *Ficus* and help improve the understanding of the physiological and morphological diversity of these plants.

*FhAG2*, a region exclusive to males in the *F. hispida* genome, is only found in the male genome and is absent in the female genome before and during maturation and the inflorescence of the female flower. But in the case of female *F. carica* species, we compared similar genes. This gene with a specific deletion, and there is a chromosomal leap between this gene in *F. carica* and *F. hispida*. A phylogenetic tree was constructed by aligning the CDS and protein sequences of this gene, the results of which were inconsistent. The CDS alignment suggested that *F. carica* evolved relatively independently, whereas according to the protein alignment, *F. carica* and *F. hispida* were more closely related. The Ka/Ks values demonstrated that the differences may be caused by the selection pressure and this protein-coding gene. The observed behavior could potentially be explained by convergent evolution within species, as *F. carica* and *F. hispida* may have developed comparable structural features to adapt to similar ecological niches. Therefor, the proteins that *Ficus* and *F. carica* share are essential for regulating *F. carica* parthenogenesis. The expression levels of this gene were increased during the development of fertilized ovules because it was not expressed in the male *F. hispida* inflorescences. As a result, the gene shared by female plants of *F. carica* may be able to both stimulate the maturation of female flowers without pollination and replace the gene’s increased expression levels, which are necessary for the pollination process in *F. carica*. The edible portions of ripe *F. carica* are the receptacles. Hence, increased expression of this gene may cause parthenocarpy in *F. carica*, and more research is required to determine the precise roles played by this gene.

*F. carica* is a species that is edible and useful in medicine since it contains a variety of bioactive chemicals. Nevertheless, the metabolic and biosynthetic pathways of these chemicals have been the subject of very few studies. The genomic, transcriptomic, and metabolomic data provided new insights into the biosynthetic processes in *F. carica*, with transcriptomic analysis revealing marked differences in the ‘signal transduction of plant hormones’ and ‘biosynthesis of secondary metabolites’. Secondary metabolite analysis showed that the ‘biosynthesis of anthocyanins’ demonstrated remarkable variations amongst the different varieties of *F. carica*. Due to the varying accumulation of anthocyanins, the four varieties of *F. carica* that were chosen for this investigation had remarkably diverse fruit colors. The ‘biosynthesis of anthocyanins’ was completed based on the flavonoid metabolic pathway, which is divided into two stages. The first stage involves *CHS*, *CHI*, *F3H*, and *F3′5′H*, it is the common pathway of flavonoid biosynthesis, and is called the pre-synthesis reaction of anthocyanin biosynthesis. The second stage involves the enzymes *DFR*, *ANS*, and *UFGT*, which are unique to anthocyanin biosynthesis, and this stage is called the late synthesis reaction of anthocyanin biosynthesis (Williams and Grayer, 2004; Petroni and Tonelli, 2011). Ten significant gene families, *PAL*, *C4H*, *4CL*, *CHS*, *CHI*, *F3H*, *F3’H*, *DFR*, *ANS*, and *UFGT* were found to be associated with the production pathway of *fig* anthocyanins in this study. The differential expression levels of genes related to anthocyanin biosynthesis were consistent with the contents of anthocyanins in *F. carica*.

In particular, the expression levels of *CHS* and *UFGT* demonstrated significant variations amongst the different varieties of *F. carica*, which was similar to potatoes (Cho et al., 2016) and jujubes (Zhang Q. et al., 2020), indicating that these genes play an essential role in the biosynthesis of anthocyanins (Wang et al., 2017). *F. carica* possessed 31 members of the *CHS* gene family, of which 13 were expressed at different levels. Additionally, we found a tandem repeat sequence that supports the *figs*’ *CHS* gene family amplification. Furthermore, different *fig* types have distinct *CHS* expression patterns. A phylogenetic tree constructed using the *CHS* proteins in *F. carica* and other species, showed that *FcCHS3, FcCHS4, FcCHS5, FcCHS6, FcCHS7, FcCHS8, FcCHS9, FcCHS10*, and *FcCHS11*, belonged to a single subfamily and the *CHS* of *F. carica* and other *Ficus* species has evolved along different trends.

The “anthocyanin biosynthesis pathway” was created using two different types of genes: regulatory genes, which control the expression patterns and levels of structural genes, and the structural genes, which encode the various essential enzymes involved in anthocyanin biosynthesis. The latter encoded transcription factors, primarily *WD40*, *bHLH*, and *MYB* (Schwinn et al., 2014). These genes are transcription factors that regulate *F. carica* biosynthesis, which is one of the main mechanisms underlying the diversity of *F. carica* fruits. One of the features of *Ficus* plants is the hidden head inflorescence. Studying the fruit diversity of this variety of *Ficus* plant, the linked genes that contribute to its diversity, and its selective preservation are crucial for future research because of its higher economic value.

In summary, the transcriptome and secondary metabolome analyses, along with the high-quality reference genome, offer valuable insights into the genome evolution and diversification of *figs*. Additionally, the data from this study offers important resources for genetic research as well as for *fig* and other *fig* plant improvement.

## Data availability statement

The datasets presented in this study can be found in online repositories. The names of the repository/repositories and accession number(s) can be found in the article/**Supplementary Material**. Theraw data generated through RNA sequencing have been deposited in the National Genomics Data Center here: "https://ngdc.cncb.ac.cn/gsub/submit/bioproject/PRJCA016877". The assembled genome was also uploaded to the National Genomics Data Center here: "https://ngdc.cncb.ac.cn/gsub/submit/bioproject/PRJCA016848.

## Author contributions

YB: Conceptualization, Data curation, Formal Analysis, Investigation, Methodology, Visualization, Writing – original draft. MH: Conceptualization, Data curation, Formal Analysis, Investigation, Methodology, Visualization, Writing – original draft. CZ: Formal Analysis, Investigation, Methodology, Writing – original draft. SJ: Formal Analysis, Investigation, Methodology, Writing – original draft. LZ: Formal Analysis, Investigation, Methodology, Writing – original draft. ZY: Conceptualization, Investigation, Methodology, Writing – original draft. QS: Methodology, Resources, Writing – original draft. ZX: Methodology, Project administration, Supervision, Writing – review & editing. MZ: Methodology, Project administration, Supervision, Writing – review & editing.
