# Supplementary figures and images for: Advancing understanding of Ficus carica: a comprehensive genomic analysis reveals evolutionary patterns and metabolic pathway insights
Source: Front Plant Sci. 2023 Dec 7;14:1298417. doi: 10.3389/fpls.2023.1298417 (PMC10754049; doi:10.3389/fpls.2023.1298417)

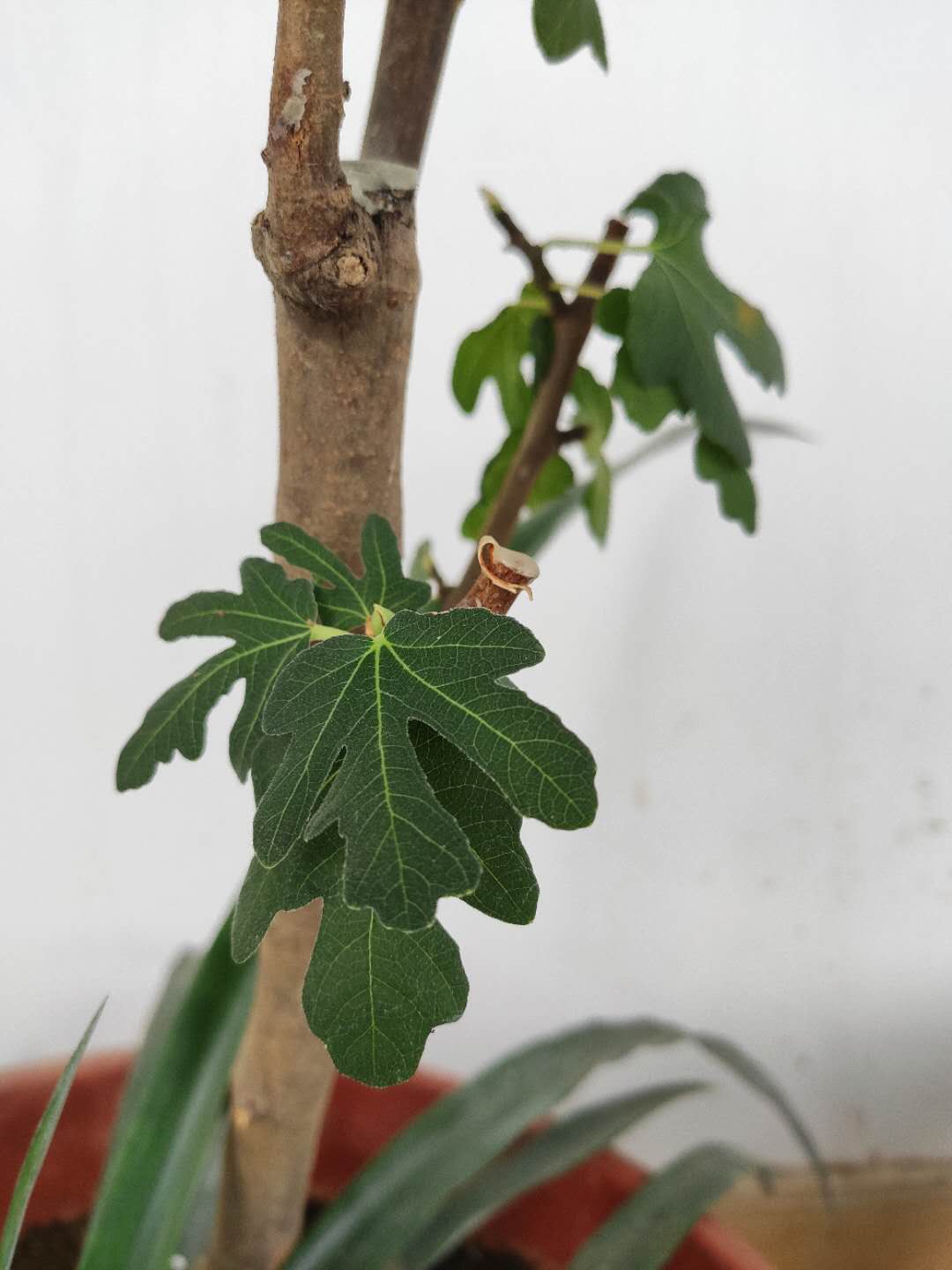

Supplement: Supplementary file 1 [file Image_1.jpeg]

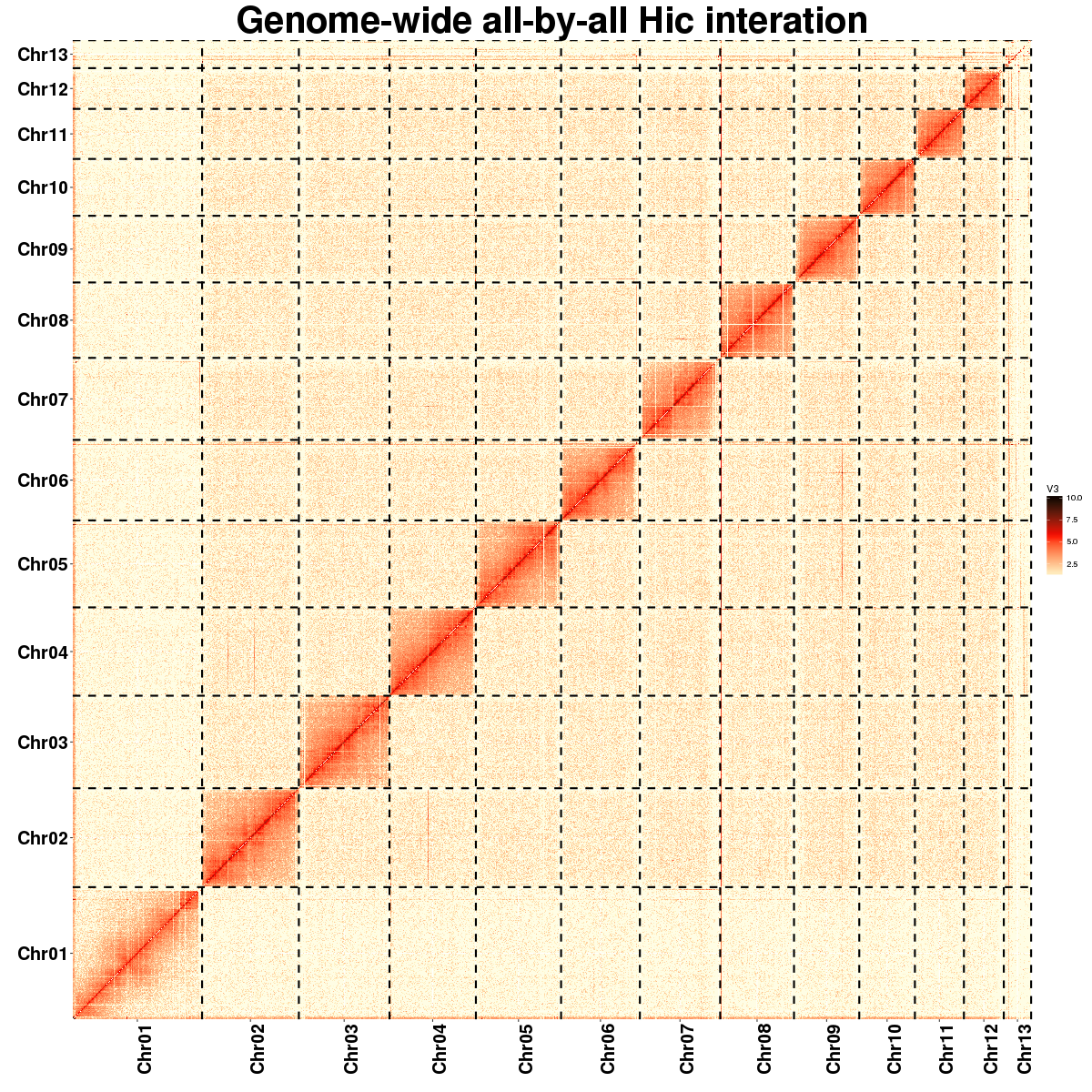

Supplement: Supplementary file 2 [file Image_2.png]

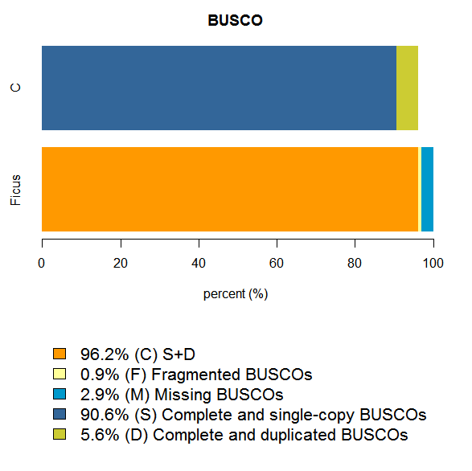

Supplement: Supplementary file 3 [file Image_3.png]

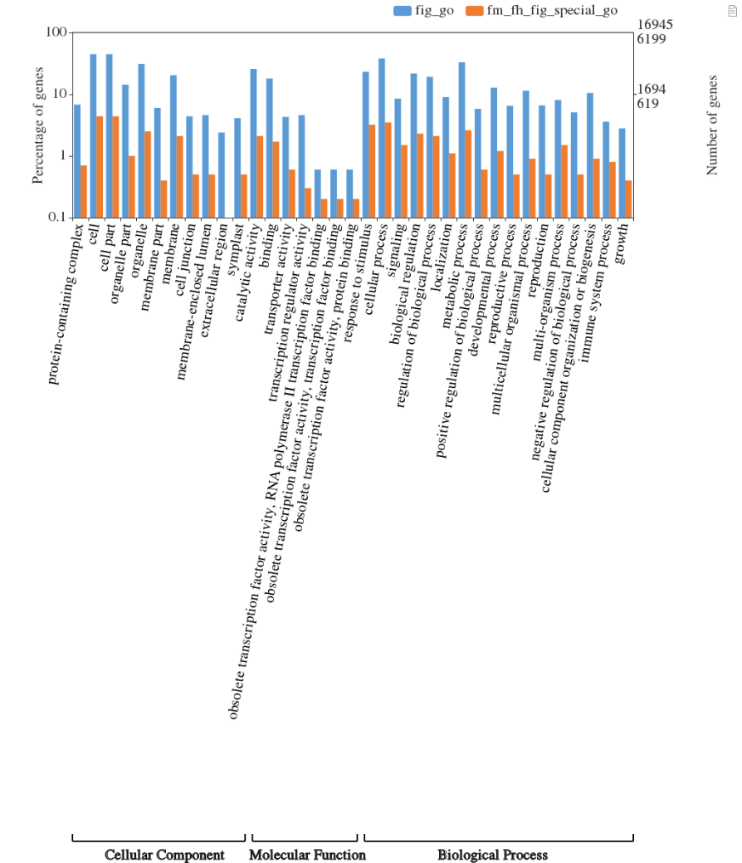

Supplement: Supplementary file 4 [file Image_4.tif]

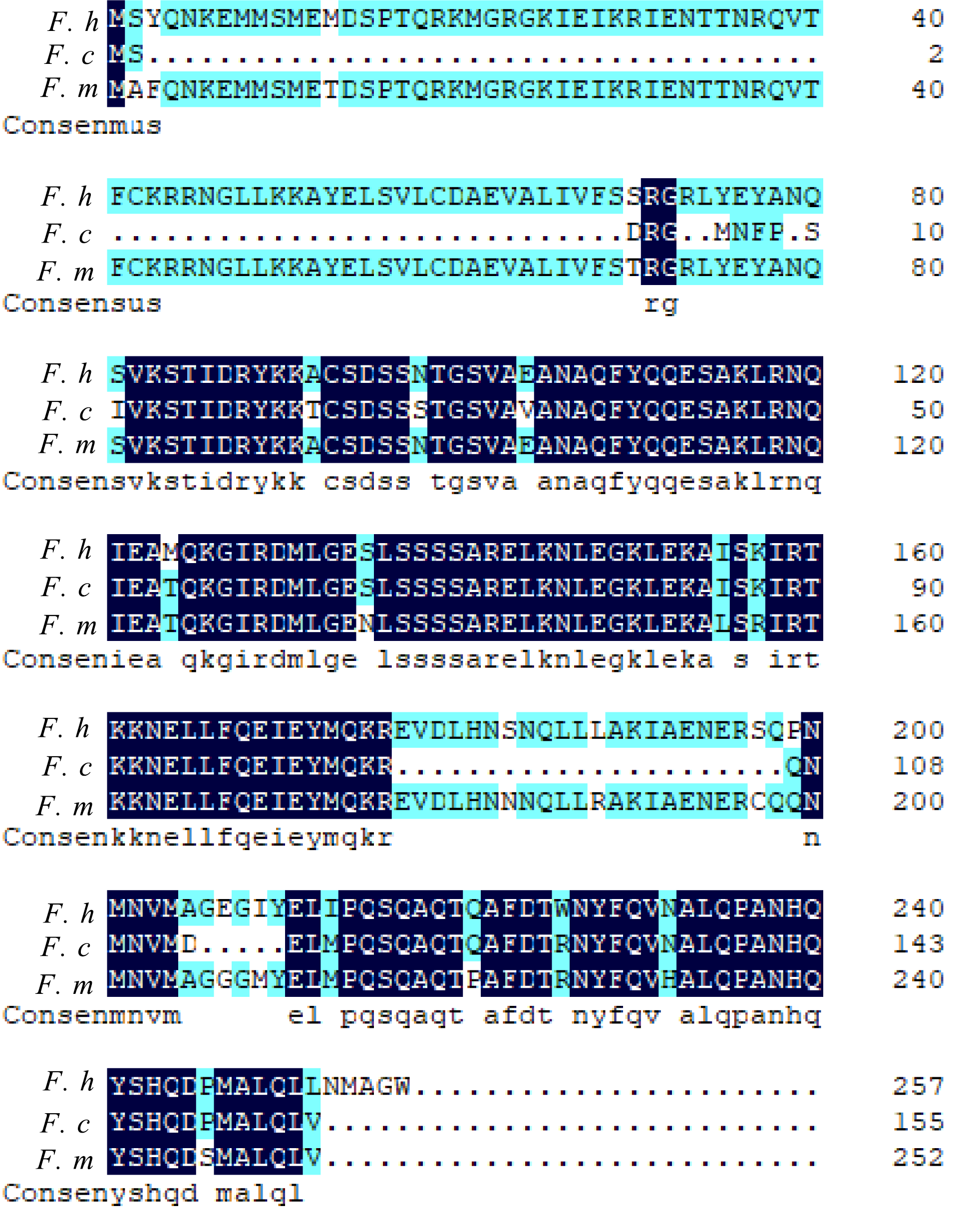

Supplement: Supplementary file 5 [file Image_5.png]

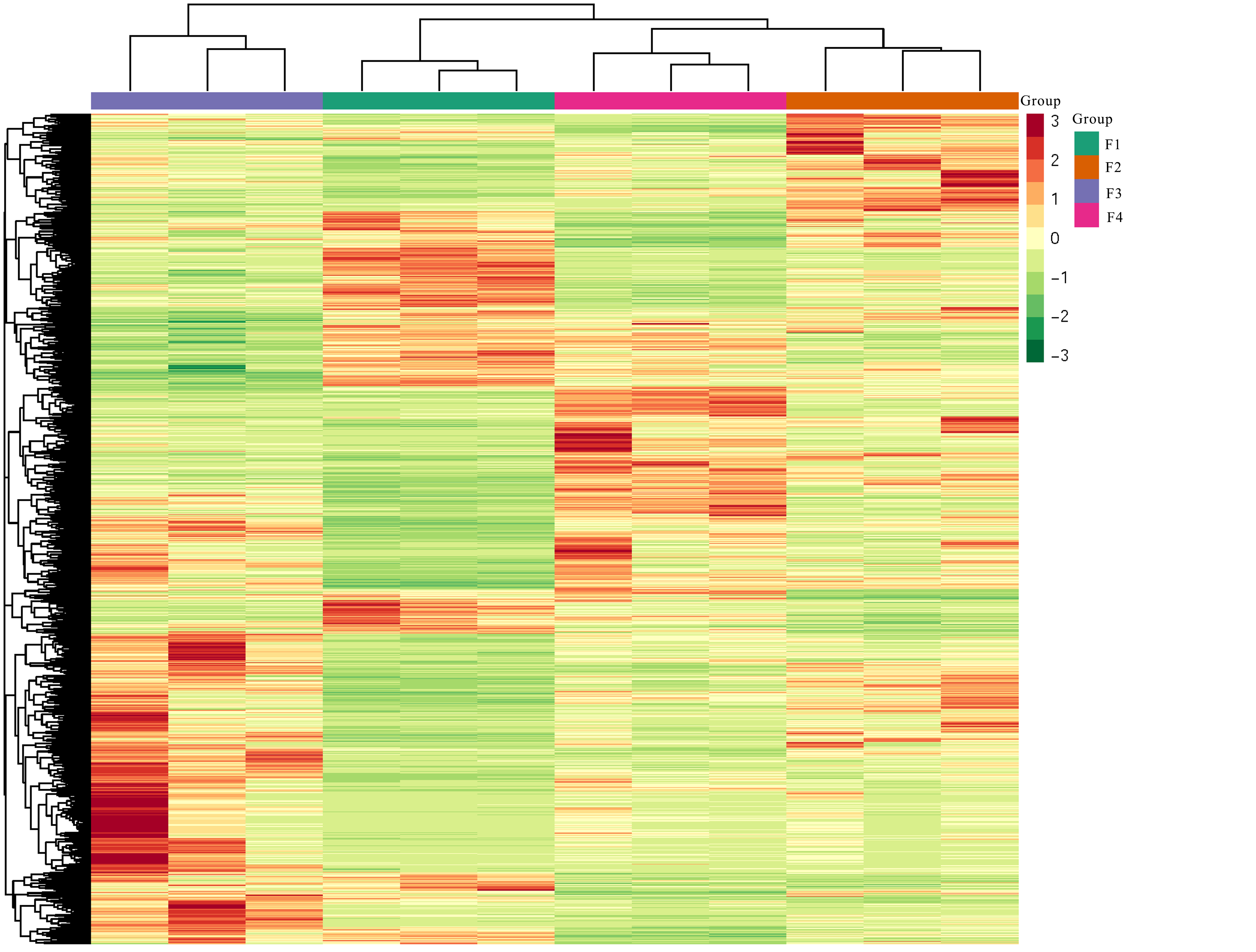

Supplement: Supplementary file 6 [file Image_6.png]

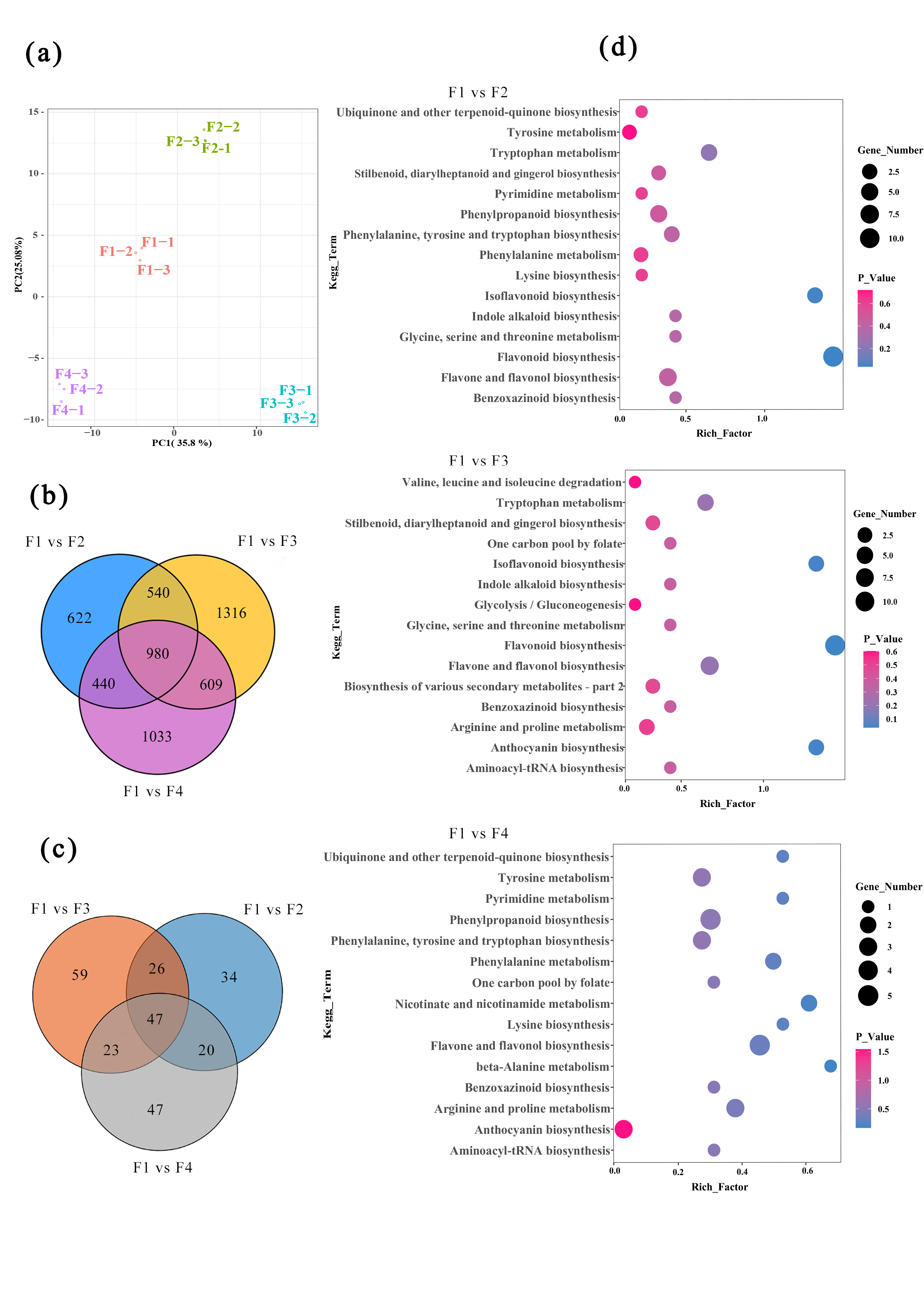

Supplement: Supplementary file 7 [file Image_7.png]

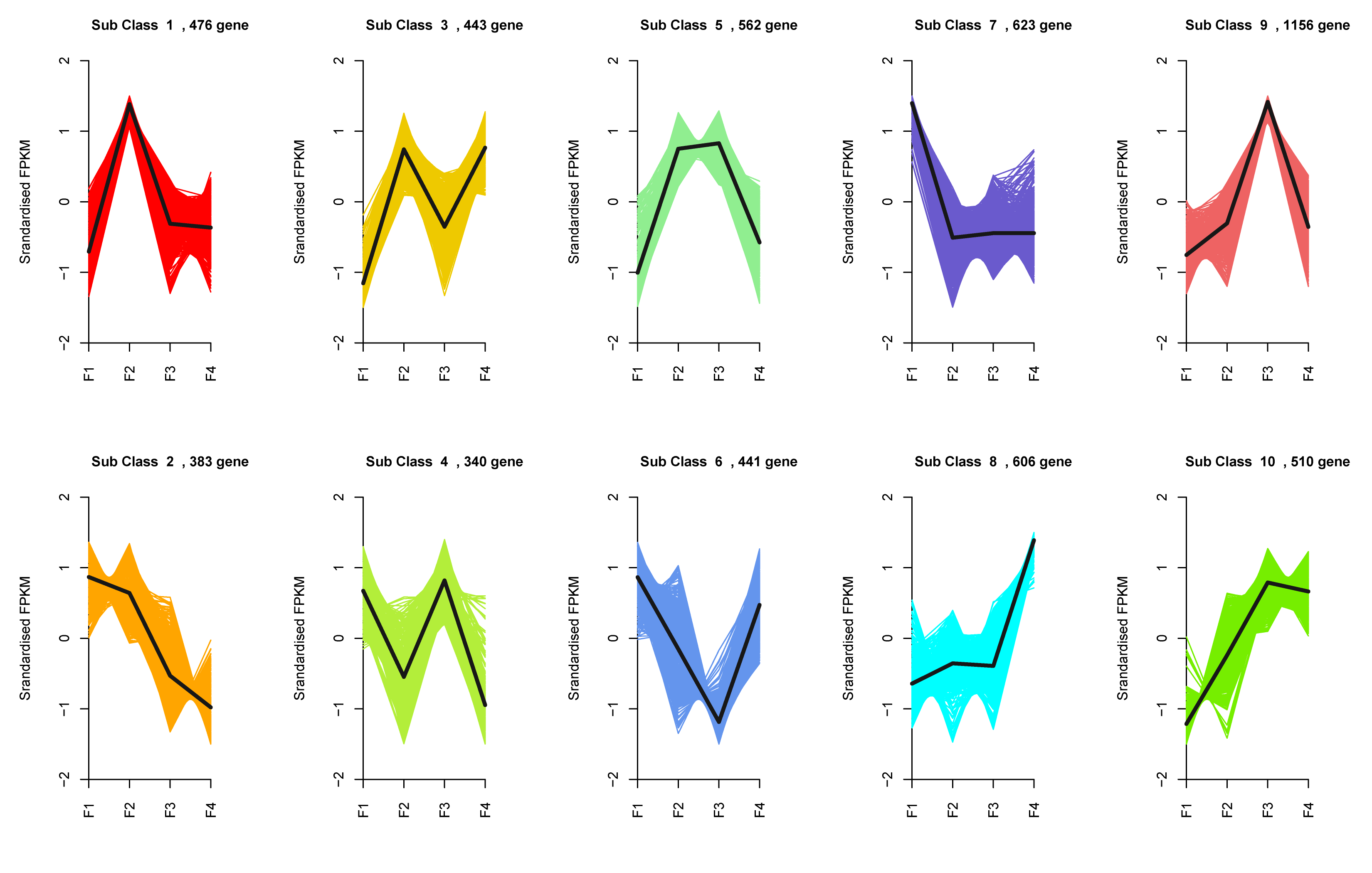

Supplement: Supplementary file 8 [file Image_8.png]

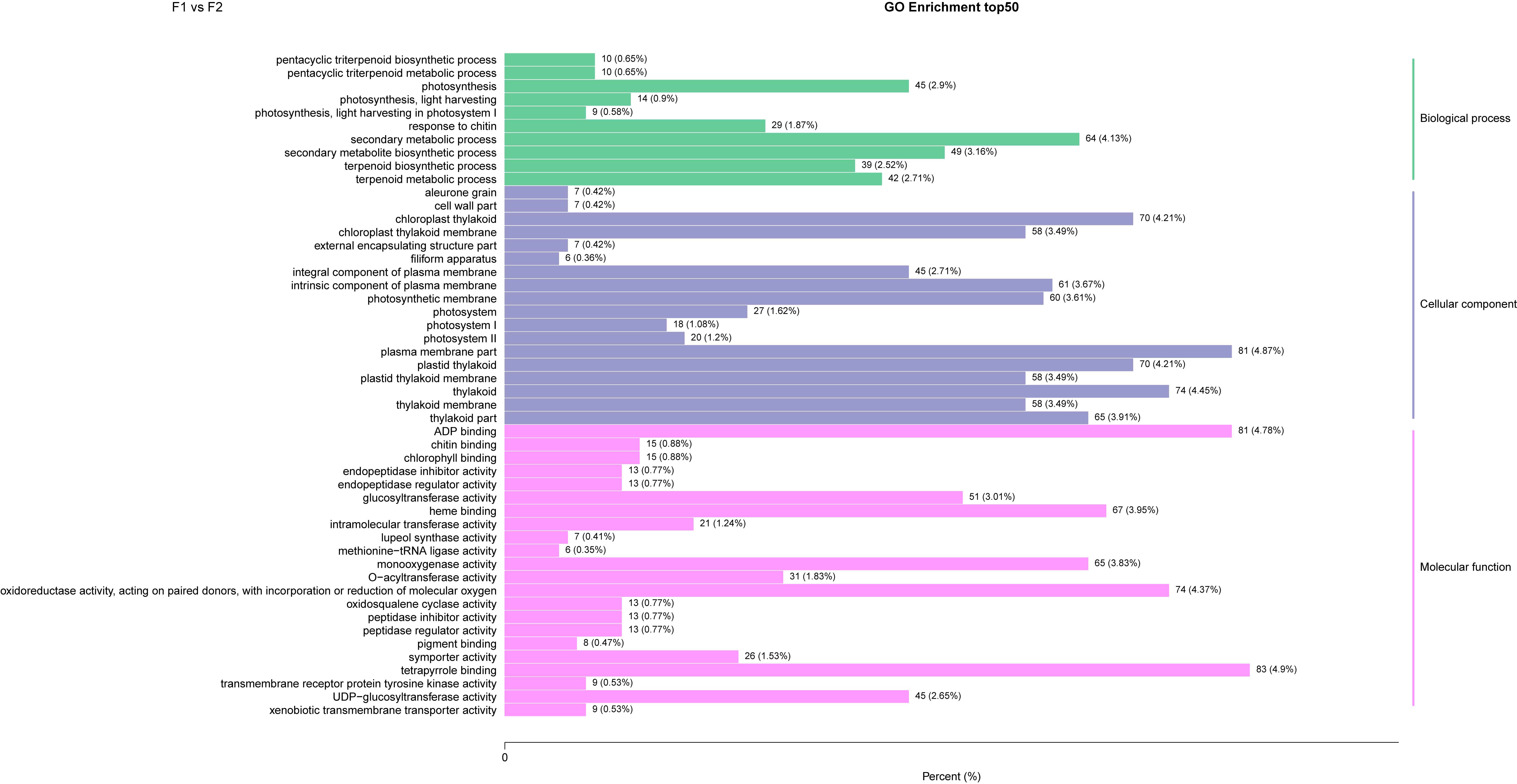

Supplement: Supplementary file 9 [file Image_9.png]

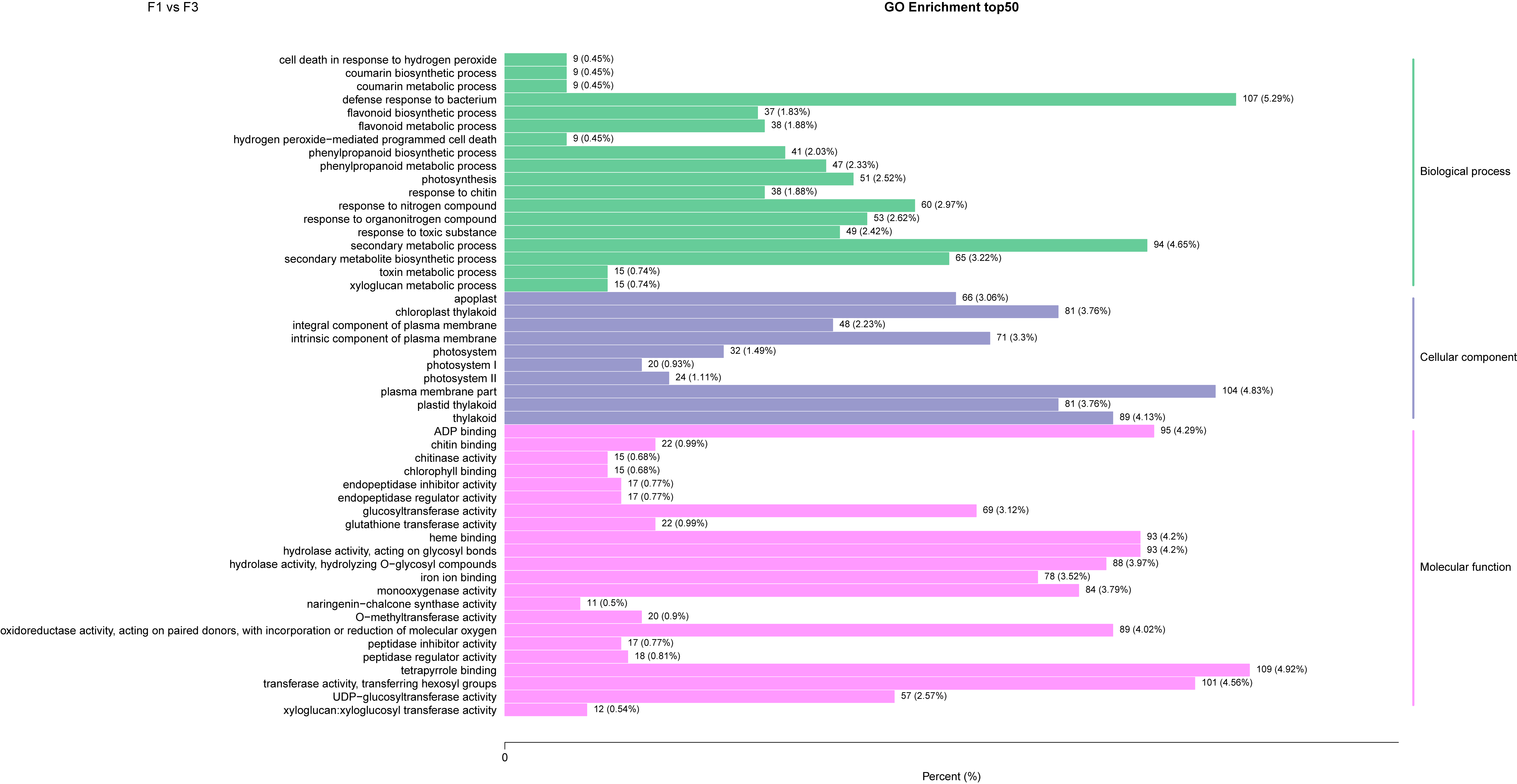

Supplement: Supplementary file 10 [file Image_10.png]

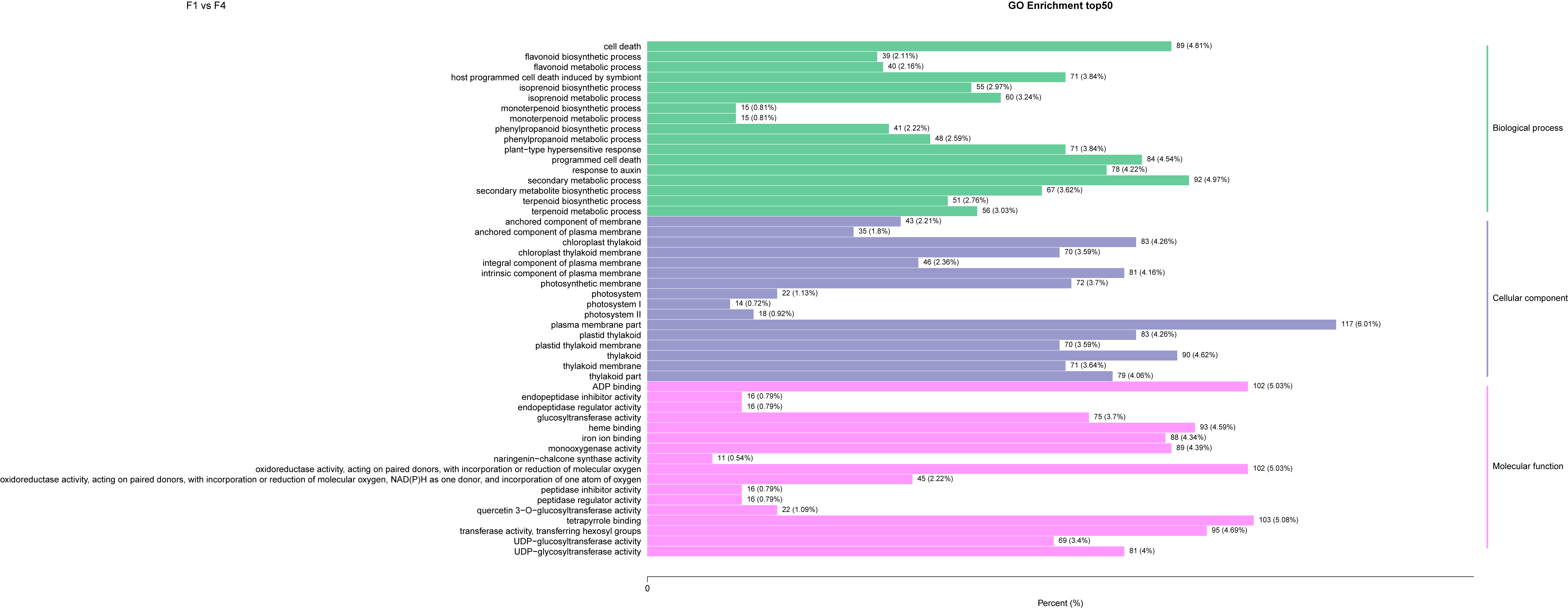

Supplement: Supplementary file 11 [file Image_11.png]

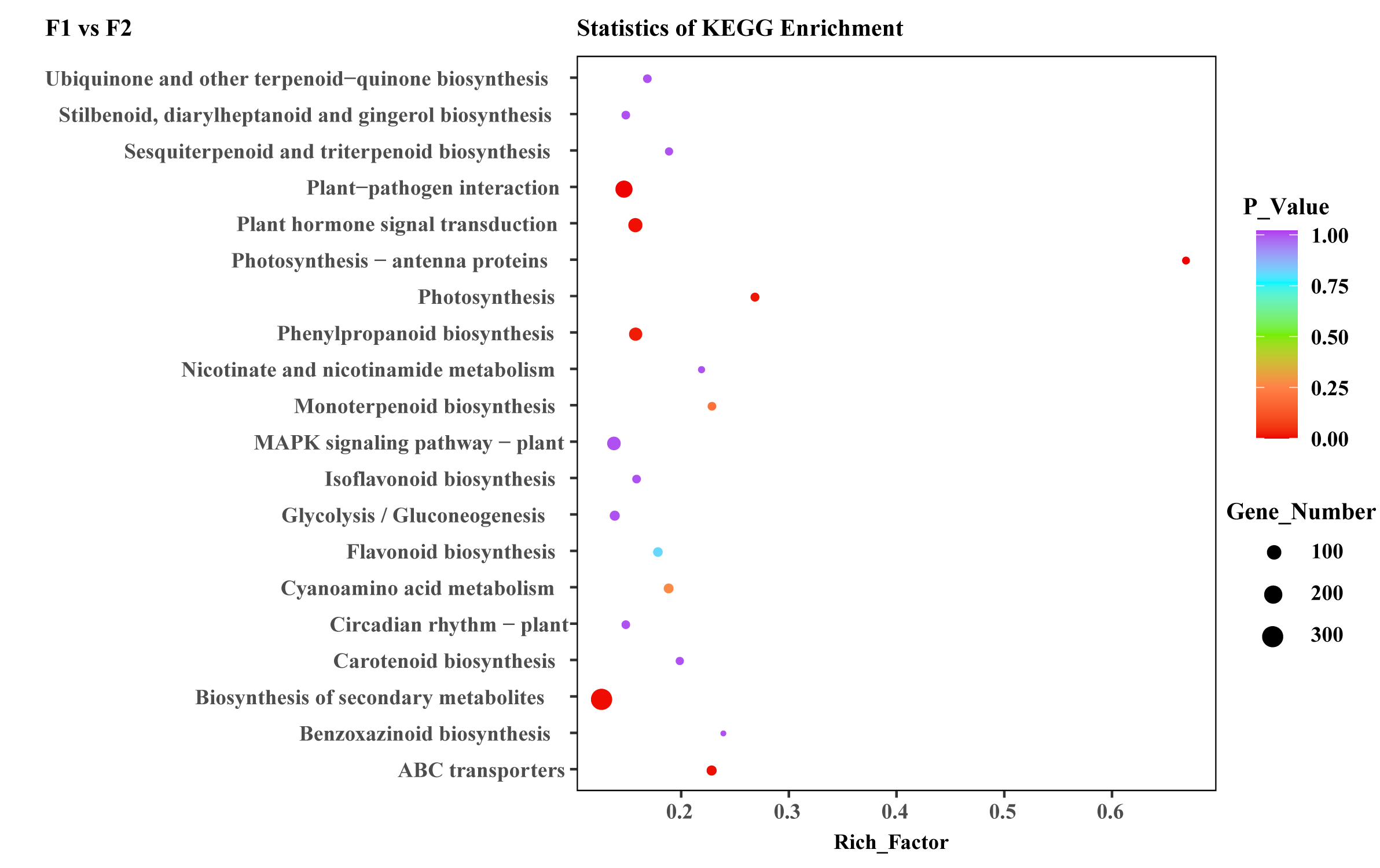

Supplement: Supplementary file 12 [file Image_12.png]

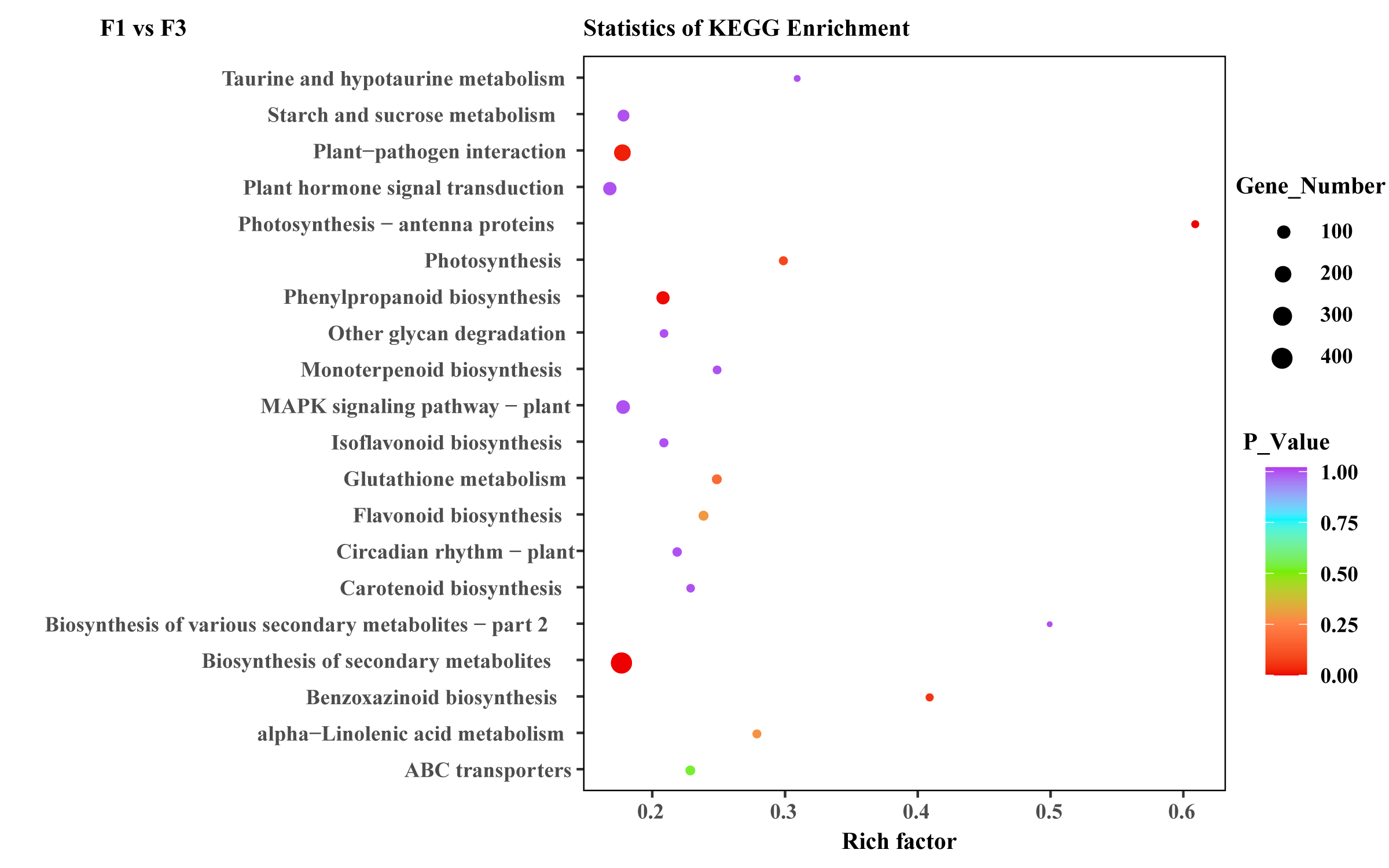

Supplement: Supplementary file 13 [file Image_13.png]

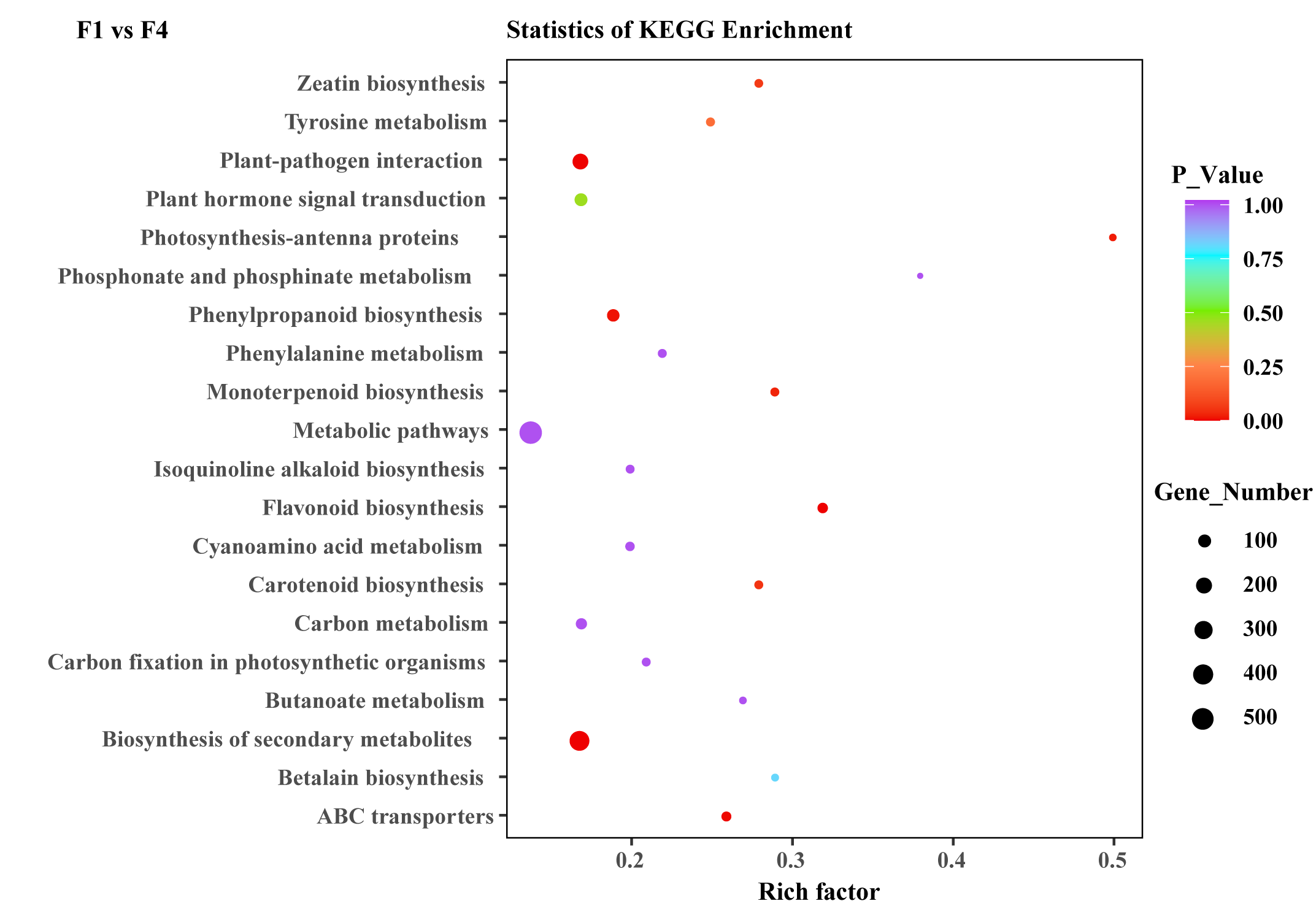

Supplement: Supplementary file 14 [file Image_14.png]
